# The plant nuclear lamina disassembles to regulate genome folding in stress conditions

**DOI:** 10.1038/s41477-023-01457-2

**Published:** 2023-07-03

**Authors:** Nan Wang, Zhidan Wang, Sofia Tzourtzou, Xu Wang, Xiuli Bi, Julia Leimeister, Linhao Xu, Takuya Sakamoto, Sachihiro Matsunaga, Andreas Schaller, Hua Jiang, Chang Liu

**Affiliations:** 1 Department of Epigenetics, Institute of Biology, University of Hohenheim, Stuttgart, Germany; 2 Department of Plant Physiology and Biochemistry, Institute of Biology, University of Hohenheim, Stuttgart, Germany; 3 Center for Plant Molecular Biology (ZMBP), University of Tübingen, Tübingen, Germany; 4 Shandong Provincial Hospital, Shandong First Medical University, Jinan, China; 5 Applied Chromosome Biology, Leibniz Institute of Plant Genetics and Crop Plant Research (IPK), Gatersleben, Germany; 6 Department of Applied Biological Science, Faculty of Science and Technology, Tokyo University of Science, Noda, Japan; 7 Department of Integrated Biosciences, Graduate School of Frontier Sciences, The University of Tokyo, Kashiwa, Japan

**Keywords:** Plant molecular biology, Genomics

## Abstract

The nuclear lamina is a complex network of nuclear lamins and lamin-associated nuclear membrane proteins, which scaffold the nucleus to maintain structural integrity. In *Arabidopsis thaliana*, nuclear matrix constituent proteins (NMCPs) are essential components of the nuclear lamina and are required to maintain the structural integrity of the nucleus and specific perinuclear chromatin anchoring. At the nuclear periphery, suppressed chromatin overlapping with repetitive sequences and inactive protein-coding genes are enriched. At a chromosomal level, plant chromatin organization in interphase nuclei is flexible and responds to various developmental cues and environmental stimuli. On the basis of these observations in *Arabidopsis*, and given the role of *NMCP* genes (*CRWN1* and *CRWN4*) in organizing chromatin positioning at the nuclear periphery, one can expect considerable changes in chromatin–nuclear lamina interactions when the global chromatin organization patterns are being altered in plants. Here we report the highly flexible nature of the plant nuclear lamina, which disassembles substantially under various stress conditions. Focusing on heat stress, we reveal that chromatin domains, initially tethered to the nuclear envelope, remain largely associated with CRWN1 and become scattered in the inner nuclear space. By investigating the three-dimensional chromatin contact network, we further reveal that CRWN1 proteins play a structural role in shaping the changes in genome folding under heat stress. Also, CRWN1 acts as a negative transcriptional coregulator to modulate the shift of the plant transcriptome profile in response to heat stress.

## Abstract (web summary)

The nuclear lamina in *Arabidopsis*, a protein meshwork beneath the nuclear envelope, disassembles under various abiotic stresses, modulating changes in three-dimensional genome organization and gene transcription in stress response.

## Main

The nuclear lamina is a layer of protein meshwork underneath the nuclear envelope, in which nuclear lamin proteins are the main constituents. Besides providing mechanical support to the nucleus, the nuclear lamina also participates in regulating many events in the nucleus, such as gene expression, chromatin organization, DNA repair and DNA replication^1^. In plants, the nuclear lamina is composed of a group of plant-specific, coiled-coil domain-containing proteins and lamin-binding membrane proteins, among which those belonging to the nuclear matrix constituent proteins (NMCPs) family were discovered first and have been investigated most extensively^2,3^. NMCPs are highly conserved in the plant kingdom, but they do not share any sequence homology with animal nuclear lamins. Amongst the NMCP(s), in each plant species studied so far, there is at least one showing preferential localization at the nuclear periphery^2,4–12^. While the sole NMCP homologue in the basal land plant *Marchantia* appears to be dispensable for vegetative growth^10^, *NMCP* genes in *Arabidopsis* are essential for plant viability^13^. Genetic crosses revealed functional redundancy of *Arabidopsis NMCP* genes, which are also named *CRWNs* (*CROWDED NUCLEI*). In an earlier study by Wang and colleagues, it was found that quadruple *crwn* mutants, as well as some triple *crwn* mutants, could not be recovered from a segregating population, indicating the requirement of a minimum level of CRWN activities to complete the plant life cycle^13^.

*Arabidopsis* CRWN1 and CRWN4 proteins are located at the nuclear periphery. Mutant plants lacking these CRWN proteins develop spherical nuclei that are noticeably smaller than those in wild-type plants^7,8^. On the other hand, overexpression of *CRWN1* promotes nuclear deformation, leading to the formation of ring-like and bleb-like structures on the nuclear envelope^14^. Transcriptomic analyses of *crwn* single and double mutants revealed that the gene expression profile of these mutants was featured with ectopic activation of pathways related to stress responses^15^. Several transcription factors and coregulators were found to interact with CRWN1, suggesting the involvement of the plant nuclear lamina in regulating gene expression^16,17^. Reminiscent of the *crwn* mutant transcriptome, mutants of lamin-interacting membrane proteins in the PNET2 family also show activated stress pathways^18^.

The *Arabidopsis* nuclear lamina selectively interacts with chromatin regions, creating a non-random chromatin distribution pattern at the nuclear periphery where repressed chromatin is enriched^19,20^. Recent work showed that CRWN1 and CRWN4 help in establishing a scattered distribution of centromeres in the nucleus^21^. At a chromosomal level, plant chromatin organization in interphase nuclei displays flexibilities. Many developmental cues and environmental factors, such as dedifferentiation^22^, leaf development^23^, seedling growth^24^, floral transition^25^, seed development^26^, light intensity^23,27^, microbial infection^28^ and temperature stress^29^, can trigger global rearrangement of chromatin, demonstrating a tight connection between the structural arrangement of chromatin and its activities. In addition, scattered evidence implies that the plant nuclear lamina might undergo active turnover^16,30^. However, given the role of *NMCP* genes (that is, *CRWN1* and *CRWN4*) in organizing chromatin at the nuclear periphery^20^, it remains unclear whether the chromatin–nuclear periphery interactions are dynamic when the global chromatin organization patterns are being altered in plants.

In this study, we show that plant nuclear lamina disassembles substantially following several types of abiotic stress treatment, including heat stress. During the heat-stress response, chromatin domains, initially tethered to the nuclear envelope, remained largely occupied by CRWN1 and become scattered in the inner nuclear space. We further show that during heat stress, CRWN1 proteins function as structural factors to guide the genome to adopt an alternative folding conformation. Also, CRWN1 acts as a negative transcriptional coregulator, modulating transcriptional reprogramming in response to heat stress.

## Results

### Changes in chromatin positioning under heat stress

**Fig. 1:**
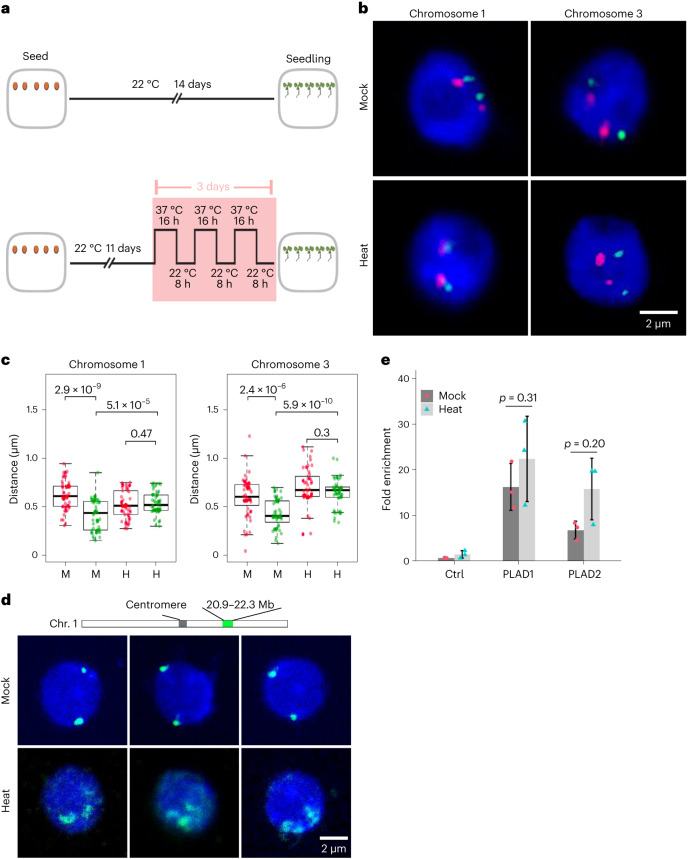
PLAD chromatin detaches from the nuclear periphery under heat stress. **a**, The experiment setup. **b**,**c**, Representative FISH images (**b**) and quantification (**c**) showing the localization of PLAD (green) and non-PLAD (red) chromatin with respect to the nuclear periphery. The box plots in **c** indicate the median (line within the box), the lower and upper quartiles (box), margined by the largest and smallest data points that are still within the interval of 1.5 times the interquartile range from the box (whiskers); outliers are not shown. For chromosome 1, *n* = 53 for mock and *n* = 50 for heat-stress treatment. For chromosome 3, *n* = 52 for mock and *n* = 48 for heat stress. The *p* values shown above the box plots were obtained from two-sided Mann–Whitney *U*-tests. M and H stand for mock and heat-stress treatment, respectively. **d**, Representative chromosome painting images showing altered localization patterns of a ~1.3 Mb genomic region after heat treatment. This probed genomic region mainly belongs to PLADs. The sketch shown above depicts the location of the probed region. **e**, ChIP–qPCR showing the interactions between CRWN1 and two PLAD loci, which overlap with gene loci *AT1G65750* (PLAD1) and *AT3G29767* (PLAD2), respectively. ‘Ctrl’ refers to a non-PLAD locus. The relative fold enrichment was calculated by using the *TUB2* genomic locus as the reference. Error bars mean standard deviation of three biological replicates; *p* values indicate results of two-sided *t*-tests. Images in **b** and **d** are representatives from two independent experiments with similar patterns. Source data

Our recent work on CRWN1 and CRWN4 indicated that they were required for establishing specific perinuclear chromatin localization patterns^20^. At the nuclear periphery, CRWN1 proteins preferentially interact with chromatin regions having low accessibility, which overlap with inactive protein-coding genes and transposons^20^. Given the fact that plants show extensive chromatin-based transcriptional reprogramming during stress responses^31^, we asked whether stress could trigger changes in the chromatin–nuclear lamina interaction patterns. Of many widely studied abiotic stress conditions, we chose heat stress in our initial experiment because of the availability of published data documenting changes in transcriptome, chromatin structure and genome organization during heat-stress acclimation^29,32^, which together implied possible dynamic chromatin positioning at the nuclear periphery. To address this question, we selected a few genomic loci belonging to plant nuclear lamina-associated domains (PLADs), and examined their localization in heat-stressed and control plants (Fig. 1a). In control plants (that is, without heat-stress treatment), fluorescent in situ hybridization (FISH) probes recognizing PLAD loci showed preferential localization near the nuclear envelope; on the contrary, such perinuclear positioning was lost in heat-stressed plants (Fig. 1b,c and Supplementary Table 1). In addition, our FISH experiment revealed a higher incidence of chromatin decondensation of the probed genomic regions in stress-treated plants, which was similar to the changes in centromeric repeats triggered by heat^29^ (Supplementary Fig. 1). We also observed similar changes in chromatin localization when FISH was applied to a larger genomic region (~1.3 megabase pairs (Mb)), which mainly belonged to PLADs^20^. Of all the nuclei that we randomly sampled (*n* > 50), over 80% from mock-treated plants showed distinct FISH signals at the nuclear periphery; whereas over 80% from heat-stressed plants showed apparent chromatin decondensation along with considerable detachment from the nuclear envelope (Fig. 1d).

Overall, these observations agree with a recent study showing global rearrangement of chromatin organization induced by heat^32^, and further imply that it involves the dissociation of PLADs from the nuclear lamina. This idea prompted us to examine the potential dynamic interactions between PLAD loci and CRWN1 by using chromatin immunoprecipitation (ChIP). The CRWN1 ChIP was performed with a *CRWN1:2HA* tagging line, in which the fusion protein between CRWN1 and a tandem HA (haemagglutinin) tag could fully rescue *crwn1* loss-of-function phenotypes^20^. We found enrichment of the selected PLAD loci by CRWN1:2HA in both control and heat-stressed plants. To our surprise, heat treatment did not weaken the interactions between CRWN1 and these loci (Fig. 1e). Since PLAD loci lost their perinuclear localization in heat-stressed plants, we proposed that the CRWN1 protein, and perhaps other components of the nuclear lamina, acquired alternative nuclear localization patterns.

### Responses of the plant nuclear lamina to stress conditions

**Fig. 2:**
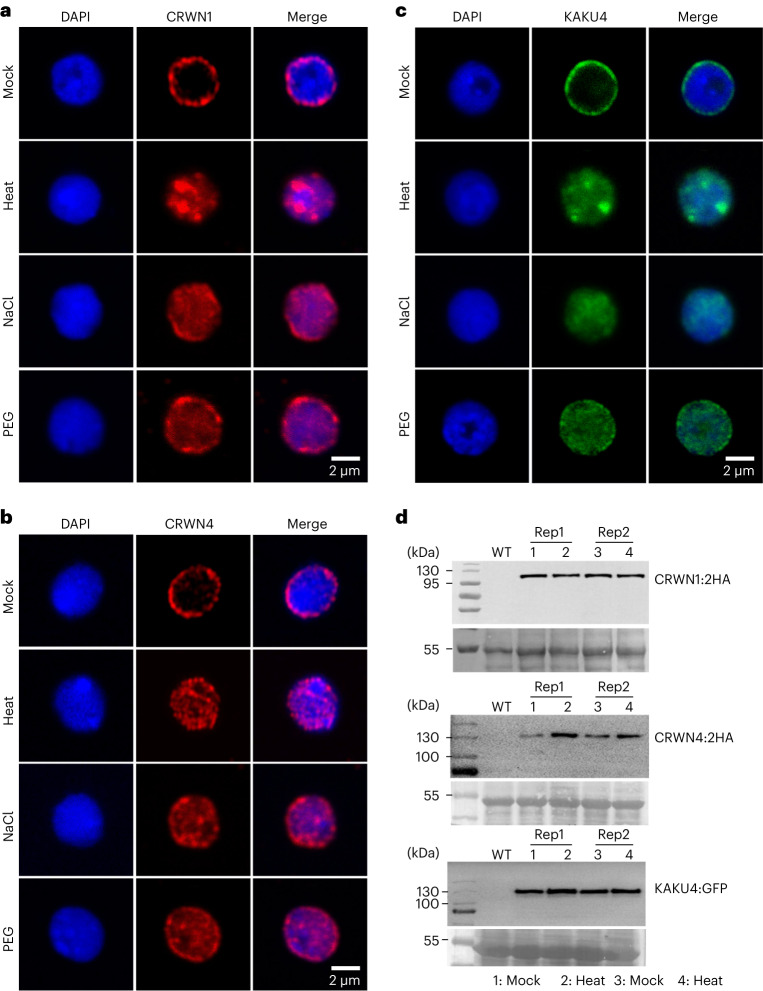
Perinuclear localization of *Arabidopsis* nuclear lamina proteins is lost under abiotic stress. **a**–**c**, Each panel shows representative confocal images of the central focal plane of nuclei. The changes in protein localization of CRWN1 (**a**), CRWN4 (**b**) and KAKU4 (**c**) in response to various abiotic stresses were observed in all randomly selected nuclei (*n* > 15) in two independent batches of experiments. ‘NaCl’ and ‘PEG’ refer to treating plant with 150 mM NaCl and 5% polyethylene glycol for two days, respectively. **d**, Plant nuclear lamina components are not cleaved or degraded under heat stress. The photographs shown below individual immunoblots depict loading control. WT indicates wild-type protein extract. Rep1 and Rep2 represent two biological replicates. Source data

**Extended Data Fig. 1:**
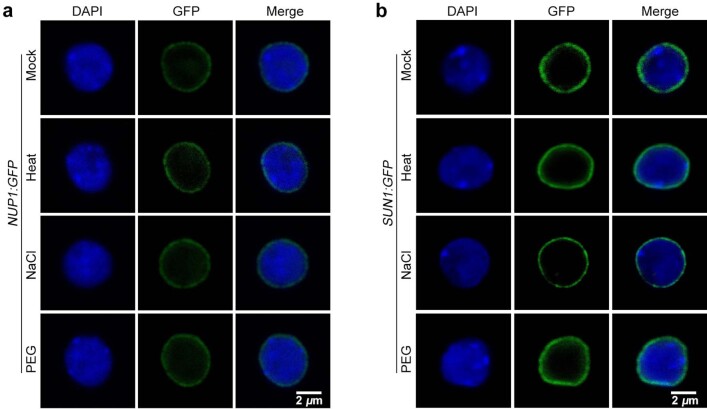
Localization of selected nuclear peripheral proteins in various growth conditions. **a, b**, Representative confocal images of NUP1:GFP (**a**) and SUN1:GFP (**b**) in nuclei isolated from plants treated with different abiotic stresses, which are described in Fig. 2. Images in **a** and **b** are representatives from two independent experiments with similar patterns.

In *Arabidopsis*, two NMCP proteins, CRWN1 and CRWN4, as well as KAKU4, are known to be involved in establishing the plant nuclear lamina^8,12,14^. To address whether the nuclear lamina acquires an alternative organization under heat stress, we examined the localization of these proteins. In total, we analysed CRWN1:2HA, CRWN4:2HA and KAKU4:GFP^14^. We also examined proteins that are located at the nuclear periphery, but are not directly involved in assembling the nuclear lamina. The selected proteins were NUP1:GFP, which was a nuclear basket protein belonging to the nuclear pore complex, and SUN1:GFP, which was an integral protein located in the inner nuclear membrane^17,33,34^. We found that nuclear lamina proteins (that is, CRWN1, CRWN4 and KAKU4) lost their specific perinuclear location during heat stress; whereas SUN1 and NUP1 did not (Fig. 2a–c and Extended Data Fig. 1). The detachment of these proteins from the nuclear periphery was not due to degradation, since protein degradation or cleavage products were undetectable (Fig. 2d). In fact, CRWN4 appeared with higher abundance after heat treatment (Fig. 2d). These findings indicate that the plant nuclear lamina disassembles during the heat-stress response, along with relocalization of its multiple components into the nuclear interior.

Previous functional studies on *Arabidopsis* nuclear lamina component genes indicated that they participated in regulating nuclear size and shape^7,8,14^. Therefore, we asked whether the disassembly of nuclear lamina during heat shock results in concomitant changes in nuclear morphology, resembling those observed in lamin mutant plants. Compared to control plants, heat-stressed plants surprisingly maintained nuclear shape and size (Supplementary Fig. 2). Statistical analyses on nuclear sizes revealed a clear difference in nuclear morphology between heat-stressed wild-type and *crwn1* plants, indicating that nuclear lamin proteins, albeit having distinct nuclear localization under heat stress, are able to maintain nuclear structure (Supplementary Fig. 2).

**Extended Data Fig. 2:**
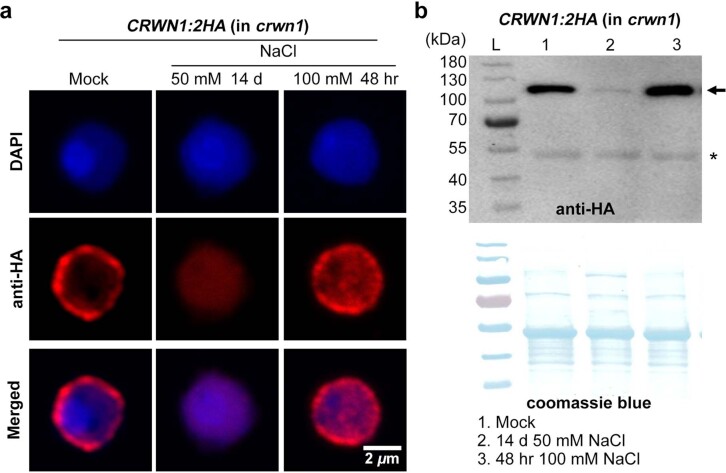
Distinct responses of CRWN1 proteins to different salt stress conditions. **a**, Immunohistostaining of CRWN1:2HA in nuclei. **b**, Immunoblots of CRWN1:2HA proteins extracted from leaves. L, protein ladder. The arrow depicts CRWN1:2HA protein signal; while the asteroid (*) marks the position of non-specific bands overlapping with a highly abundant Rubisco subunit, which is revealed with Coomassie blue staining in the bottom panel. Images in **a** and **b** are representatives from two independent experiments with similar patterns.

We further asked if other stress conditions were able to trigger similar changes to the nuclear lamina. By treating plants with salt or osmotic stresses, we also observed remarkable detachment of CRWN1, CRWN4 and KAKU4 from the nuclear periphery (Fig. 2a–c). On the contrary, SUN1 and NUP1 retained their perinuclear localization under these stress conditions (Extended Data Fig. 1). Interestingly, after prolonged stress exposure, for example, in seedlings continuously facing 50 mM NaCl stress from onset of germination, the abundance of CRWN1 dropped and the remaining protein was distributed throughout the nucleoplasm (Extended Data Fig. 2). Taken together, these results reveal the dynamic nature of the *Arabidopsis* nuclear lamina; upon perceiving different stress conditions, it disassembles and releases its component proteins into the nuclear interior.

### Dynamic lamin–chromatin interactions under heat stress

**Fig. 3:**
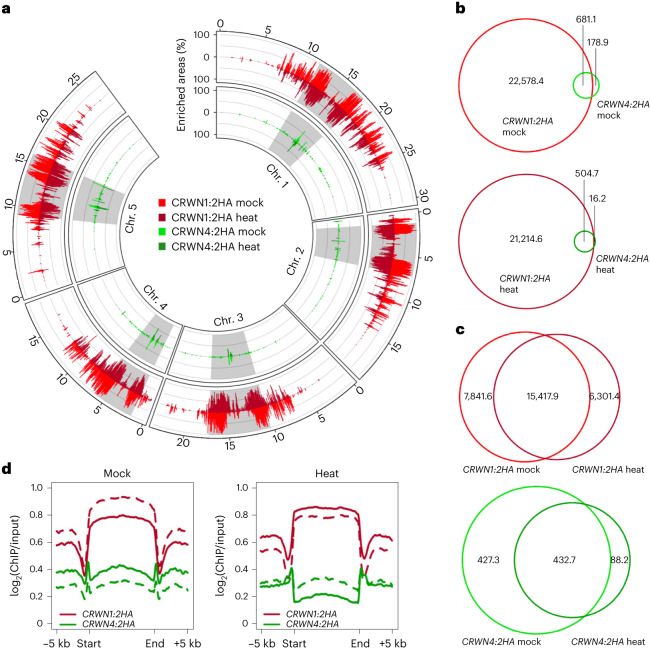
A global view of CRWN–chromatin association under heat stress. **a**, Genomic overview of CRWN1- and CRWN4-enriched chromatin. The grey blocks depict pericentromeric regions. **b**,**c**, Venn diagrams of genomic regions (unit, kb) enriched in individual samples. **b**, The amount of enriched regions shared by CRWN1 and CRWN4 under the same growth condition. **c**, The amount of regions enriched by CRWN1 (top) or CRWN4 (plot) in different growth conditions. **d**, ChIP–seq signals of CRWN1 and CRWN4 proteins across CRWN1-enriched genomic regions. The solid and dashed lines represent two biological replicates.

**Extended Data Fig. 3:**
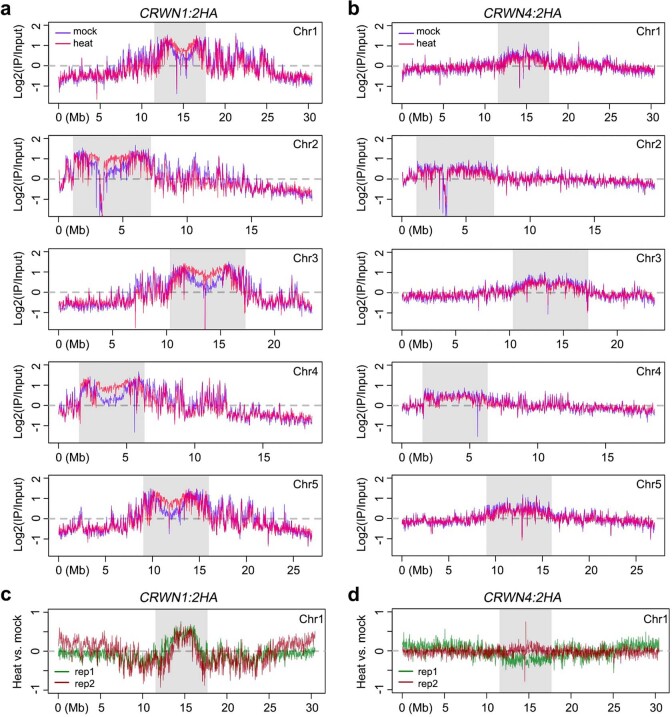
Comparison of interaction patterns between CRWNs and chromatin. **a**, **b**, Genome-wide view of CRWN1:2HA (**a**) and CRWN4:2HA (**b**) ChIP-seq signals. The grey block in each plot depicts centromeric and pericentromeric regions. All the plots were generated with a 20 kb window. **c**, **d**, Comparison of CRWN chromatin interactions with and without heat stress. The y-axis represents log_2_-transformed ratio of ChIP-seq signals.

Following up on our findings that PLAD chromatin remained in direct contact with CRWN1 proteins after the disassembly of the nuclear lamina in heat response (Figs. 1 and 2), we sought to gain a better view on the global interactions between chromatin and CRWNs in this process. To this end, we performed ChIP–seq experiments by using the *CRWN1:2HA* and *CRWN4:2HA* tagging lines in their respective mutant genetic backgrounds (as mentioned in the previous section). The *CRWN4:2HA* tagging line carried a genomic *CRWN4* sequence fused with tandem HA repeats, which could fully rescue the nuclear morphology phenotypes (smaller and round nuclei) in *crwn4* (Supplementary Fig. 3). Principal component analysis of ChIP–seq sample reads distribution patterns revealed noticeable changes in CRWN1–chromatin interactions before and after heat stress, but not in CRWN4:2HA–chromatin interactions (Supplementary Fig. 4). In addition, close examination of ChIP–seq signals across individual chromosomes indicated that CRWN1–chromatin interactions were much more prominent compared to CRWN4 (Fig. 3a, Extended Data Fig. 3 and Supplementary Data 1). The majority of CRWN4:2HA-bound chromatin, which was also enriched with CRWN1:2HA, was located in centromeric and pericentromeric regions (Fig. 3b). We also observed that heat stress influenced CRWN1– and CRWN4–chromatin interactions differentially. Compared to control plants, heat-stressed plants showed stronger CRWN1:2HA–chromatin interactions in pericentromeric regions, but the interactions in chromosome arms became weaker (Fig. 3a and Extended Data Fig. 3c). Such a shift of CRWN1:2HA–chromatin interaction patterns suggests that the decondensation of pericentromeric regions, triggered by heat stress, promotes the formation of new contacts with CRWN1:2HA. A similar amount of genomic regions were enriched with CRWN1 before and after heat stress (23,259.5 kilobase pairs (kb) in control versus 21,719.3 kb under heat), in which 15,417.9 kb were shared, and comparable amounts of regions were annotated as gain- and loss-of-enrichment (7,841.6 kb and 6,301.4 kb, respectively) (Fig. 3c). On the contrary, for CRWN4:2HA, heat resulted in much more loss-of-enrichment than gain-of-enrichment regions. Of the 860 kb CRWN4:2HA-enriched regions in control plants, 427.3 kb were lost in heat-stressed plants; while the regions that gained enrichment were 88.2 kb (Fig. 3c). Interestingly, CRWN4:2HA–chromatin interactions were stronger at the boundary of CRWN1:2HA-enriched regions (Fig. 3d), implying that CRWN1 is the main NMCP protein involved in forming nuclear lamina–chromatin contacts at the nuclear periphery. Altogether, our results indicate that *Arabidopsis* lamin–chromatin interactions largely remain under heat-stressed conditions along with the disassembly of the nuclear lamina.

### CRWN1–chromatin interactions mediate gene expression control

**Fig. 4:**
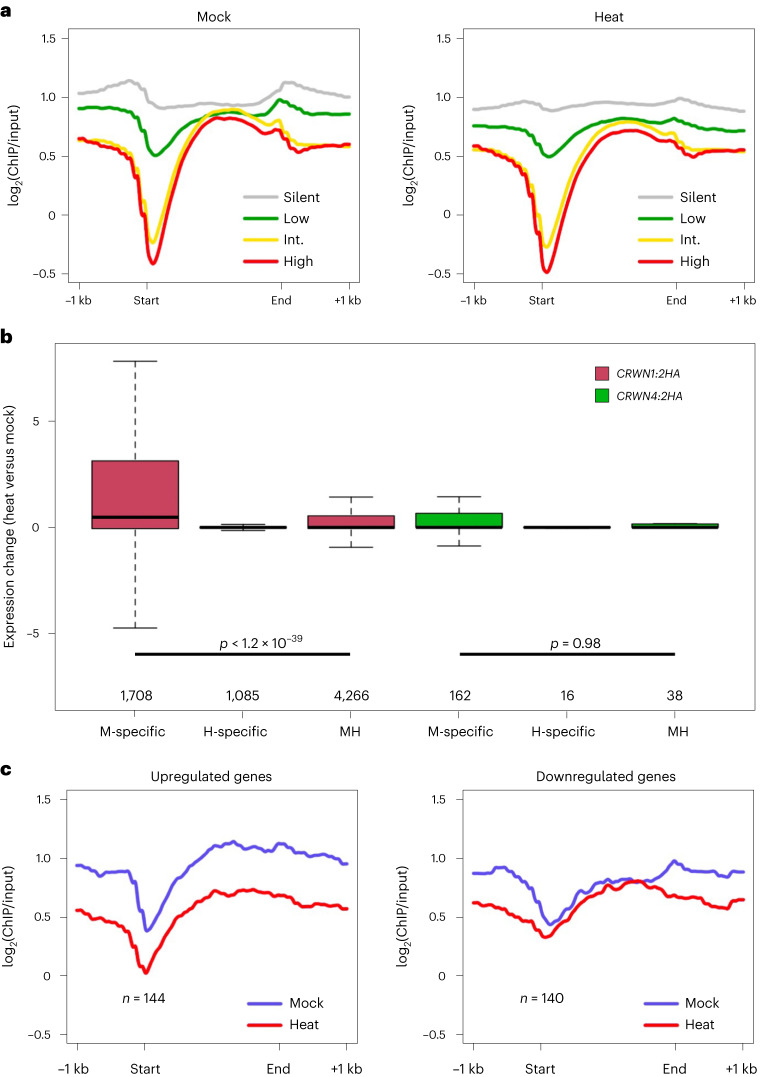
CRWN1–chromatin interactions and gene expression changes during heat-stress response. **a**, For CRWN1 target genes, their interaction profile with CRWN1 is shown according to gene expression levels. ‘Int.’ stands for the intermediate level; ‘start’ and ‘end’ depict transcription start site and transcription termination site, respectively. **b**, Changes of gene expression under heat stress. Genes enriched by CRWN only in control or heat-stressed plants are labelled as ‘M-specific’ or ‘H-specific’, respectively. Genes enriched in both conditions are labelled as ‘MH’. The *y* axis depicts differences in reads per kilobase million. The box plots indicate the median (line within the box), the lower and upper quartiles (box), margined by the largest and smallest data points that are still within the interval of 1.5 times the interquartile range from the box (whiskers); outliers are not shown. *p* values indicate the two-sided Mann–Whitney *U*-test results. **c**, CRWN1–chromatin interactions across differentially expressed genes in the ‘MH’ group defined in **b**. Source data

**Extended Data Fig. 4:**
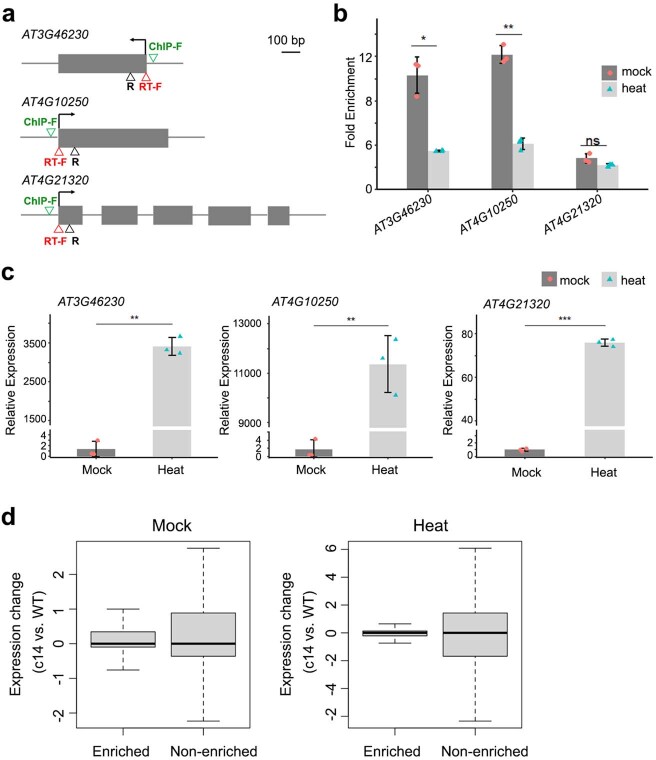
Relation between CRWN1-chroamin contacts and gene expression. **a–c**, Dynamic CRWN1-chromatin interactions and gene expression at selected loci. **a**, Schematic structures of *AT3G46230*, *AT4G10250* and *AT4G21320* showing the regions amplified by primers used for ChIP-qPCR and RT-qPCR analyses. Grey boxes represent exons. Arrows indicate transcription start sites. **b**, ChIP-qPCR analysis of CRWN1 enrichment at these gene loci under mock and heat conditions. The relative fold enrichment was normalized to an internal control locus *TUB2*, which does not interact with CRWN1 under either mock or heat conditions. The values shown are means ± SD of three independent replicates, *p < 0.05, ** *p* < 0.01, ‘ns’ indicates no significance (two-sided t-test). *p* values from left to right: 0.0184, 0.00146, and 0.092. **c**, RT-qPCR analysis showing the mRNA levels of these loci under mock and heat conditions. The relative expression levels were calculated by using *ACTIN2* as control. The values indicate means ± SD of three independent replicates, ** *p* < 0.01, *** *p* < 0.001 (two-sided t-test). *p* values from left to right: 0.0014, 0.0034, and 0.00014. **d**, Changes in gene expression in *crwn1 crwn4* mutants. The boxplots show the difference of gene expression, measured in rpkm, between WT (wild-type) and crwn14 (*crwn1 crwn4*). For each growth condition, genes are separated into two groups according to CRWN1 ChIP-seq data. The box plots indicate the median (line within the box), the lower and upper quartiles (box), margined by the largest and smallest data points that are still within the interval of 1.5 times the interquartile range from the box (whiskers); outliers are not shown. In the mock panel, n = 5974 for ‘enriched’; n = 21469 for ‘non-enriched’. In the heat panel, n = 5351 for ‘enriched’; n = 22092 for ‘non-enriched’. Two-sided Mann-Whitney U test results comparing the two boxplots indicated a *p*-value larger than 0.05. Source data

Next, we sought to explore whether dynamic CRWN1:2HA–chromatin interactions are associated with specific features of gene expression. Under both control and heat-stress conditions, CRWN1:2HA showed a negative association with gene expression (Fig. 4a). Among all the genes bound by CRWN1:2HA, silenced or weakly expressed genes exhibited stronger ChIP–seq signals than highly expressed ones, and the differences in ChIP–seq signal strength were located around the 5′ end of genes (Fig. 4a). This observation is in line with our previous report showing that CRWN1–chromatin interactions are negatively correlated to chromatin accessibility, which was a universal feature of the 5′ end of actively expressed genes^20^. We also analysed expression changes of genes, which showed dynamic changes in CRWN–chromatin interactions under heat stress. According to the annotated chromatin domains enriched by CRWN1 under mock (M) and heat (H) conditions, we refer to genes overlapping with them as ‘M-’ or ‘H-specific’ or ‘MH’ (Supplementary Data 1). Compared to genes bound by CRWN1:2HA in both conditions (that is, MH), those losing their interactions during heat stress (that is, M-specific) tended to show elevated expression (Fig. 4b). Among those M-specific genes, 57 and 34 showed significant up- and downregulation under heat stress, respectively (Supplementary Data 2). Of the 57 upregulated genes, three (*AT3G46230*, *AT4G10250* and *AT4G21320*) were annotated as closely linked to heat response. The upregulation of these three genes under heat stress was confirmed by reverse transcription quantitative real-time PCR (RT–qPCR), and ChIP–qPCR experiments confirmed substantial decrease in CRWN1–chromatin interactions at two of the three loci (Extended Data Fig. 4a–c). Similarly, of the genes in the MH group, those annotated as upregulated differentially expressed genes (DEGs) showed attenuated interactions with CRWN1:2HA under heat stress (Fig. 4c). These results reveal a correlation between the dissociation of CRWN1 from chromatin and gene upregulation during the heat-stress response. We next asked whether CRWN1/4 target genes could be differentiated from non-target genes according to transcriptional activities. However, by comparing changes in gene expression, we did not observe any notable difference between these two groups of genes (Extended Data Fig. 4d). Furthermore, CRWN1/4 target genes did not show a systematic shift towards up- or downregulation in *crwn1* *crwn4* mutants, implying that CRWN1/4–chromatin interactions alone are not sufficient to drive gene expression change (Extended Data Fig. 4d). It is also possible that the complete loss of CRWN1 and CRWN4 proteins in the double mutant plants led to the establishment of new networks among chromatin and other nucleoskeleton proteins (for example, CRWN2 and CRWN3), masking gene expression changes associated with dynamic CRWN1/4–chromatin interactions in wild-type plants.

### CRWNs mediate chromatin organization under heat stress

**Fig. 5:**
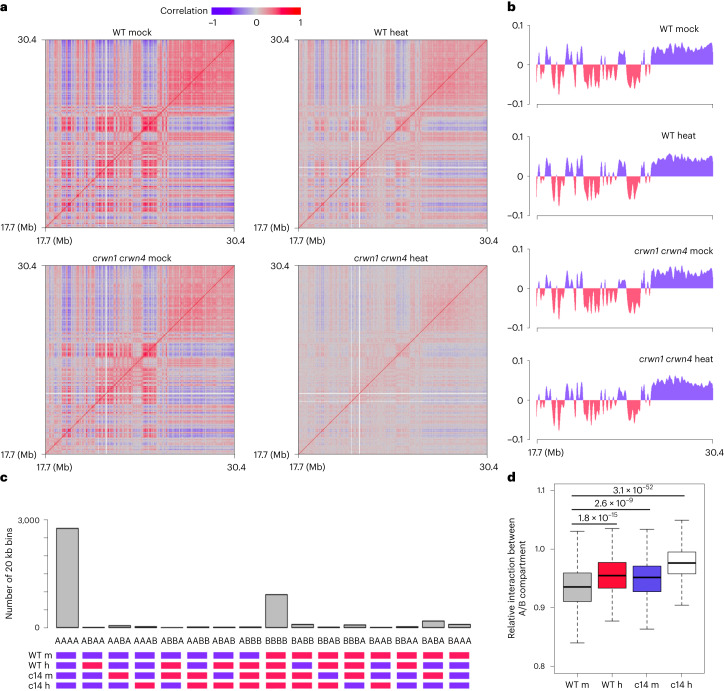
Chromatin compartments are weakened by heat and loss of CRWN1 and CRWN4. **a**, Correlation matrices of Hi-C maps of chromosome 1 right arm. **b**, Binary annotation of compartment A/B at chromosome 1 right arm. The *y* axis shows values of the eigenvector of the first component. Regions coloured in blue and red depict compartment A and B, respectively. **c**, Genome-wide A/B compartment annotation among different samples. c14, *crwn1* *crwn4*; m, mock; h, heat. **d**, Distance-normalized interactions between compartment A and B relative to the average. Sample labels are as in **c**. Chromatin contacts within 1 Mb are included in the calculation. The box plots indicate the median (line within the box), the lower and upper quartiles (box), margined by the largest and smallest data points that are still within the interval of 1.5 times the interquartile range from the box (whiskers); outliers are not shown. *n* = 400 for individual box plots. *p* values indicate the two-sided Mann–Whitney *U*-test results. Source data

**Extended Data Fig. 5:**
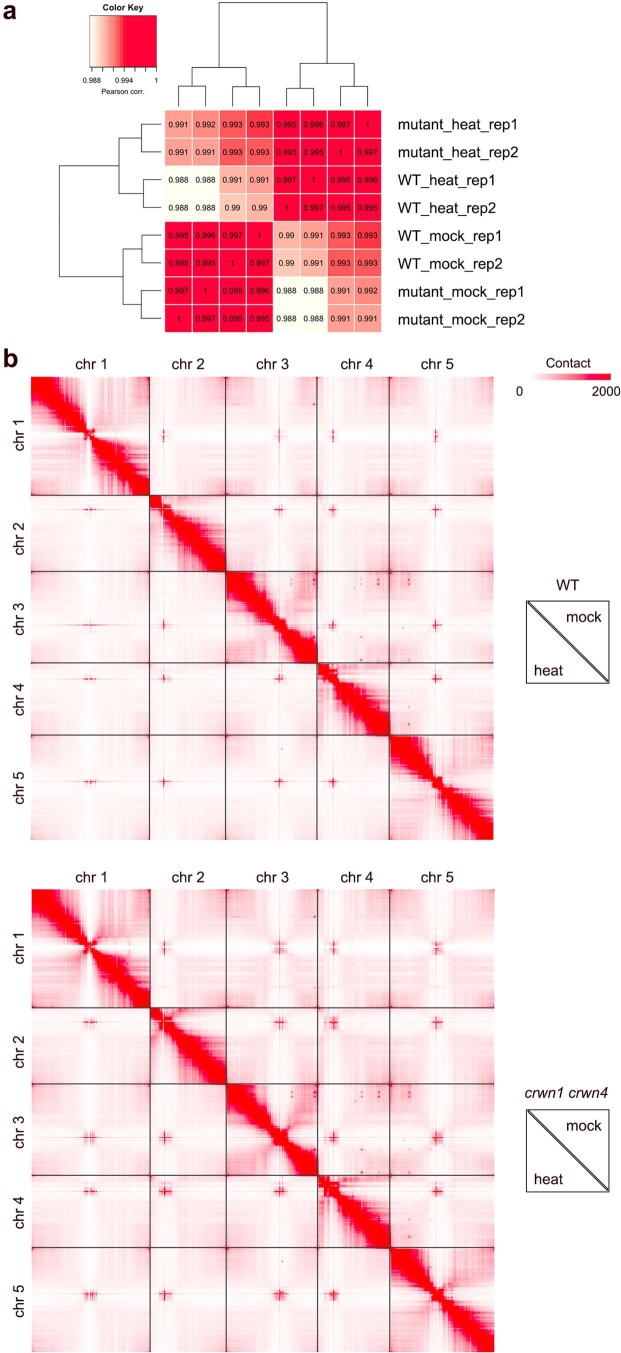
Hi-C contact maps of wild-type and *crwn1 crwn4* mutant. **a**, Pairwise comparison of Hi-C data normalized with 20 kb bins. The dendrogram shows hierarchical clustering based on Euclidean distance. Mutant and WT stands for *crwn1 crwn4* and wild-type, respectively. Numbers in individual cells indicate Pearson correlation coefficient. **b**, Genome-wide chromatin contact maps normalized with 20 kb bins.

**Extended Data Fig. 6:**
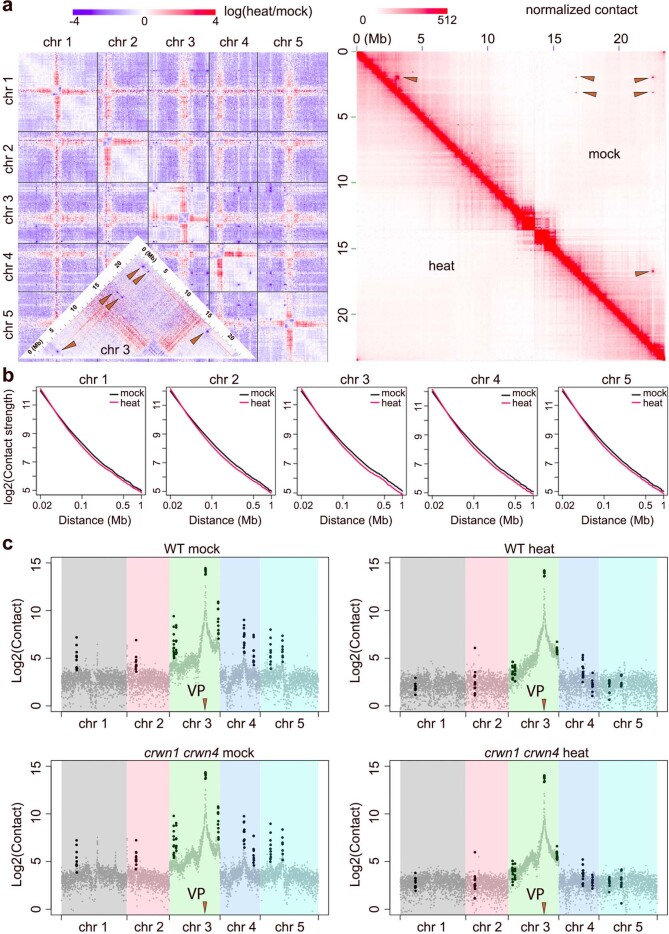
Heat stress largely weaken chromatin contact in PLAD and KEE regions. **a**, Comparison of genome-wide wild-type Hi-C contacts (left) and those at chromosome 3 (right). Colors in the left panel indicate the relative changes in chromatin interaction strengths, expressed as the ratio of heat-stressed over mock sample. The inlet highlight chromosome 3. The right panel shows chromosome 3 chromatin contact strengths. The arrowheads highlight interactions between chromosome 3 KEE regions, which are suppressed in heat-stressed plants. **b**, Comparison of chromatin organization of PLAD regions. Each panel shows chromatin interaction strength of PLAD at one chromosome. The curves describe how fast *cis*-chromatin contacts decrease as a function of genomic distance. **c**, Contact patterns of a KEE region in chromosome 3 with the entire genome, whose location is indicated with an arrow head (VP, view point). In each panel, KEE regions are highlighted with black dots.

The *Arabidopsis* CRWN1 and CRWN4 have recently been shown to modulate perinuclear chromatin positioning and pericentromere structure^20,21^. We applied the Hi-C method for genome-wide detection of chromatin interactions to examine whether CRWN1 and CRWN4 play a structural role in mediating the changes of chromatin organization under heat stress^32^. In total, Hi-C maps of wild type and *crwn1* *crwn4* under control and heat-stress conditions were generated (Fig. 5 and Extended Data Fig. 5). In line with recent studies, we observed a reduction of chromatin contacts within pericentromeres as well as reduction of many distal intrachromosomal contacts in heat-stressed wild-type plants (Extended Data Fig. 6a). Under heat stress, PLADs exhibited reduced intrachromosomal contacts (Extended Data Fig. 6b), which agrees with our observation from FISH that such a stress condition resulted in the dispersal of PLADs in nucleoplasm and reduction of chromatin contact strength among them (Fig. 1d). Consistent with a recent report by Sun and colleagues, as shown in Extended Data Fig. 6a, we also observed that heat stress caused a remarkable reduction of contact amongst KNOT ENGAGED ELEMENTS (KEEs), which had strong intra- and interchromosomal interactions^32,35^. A closer examination of KEEs in all Hi-C maps revealed that KEE patterns were highly similar in wild type and *crwn1* *crwn4* under the same growth condition, suggesting that CRWNs do not play a structural role in these genomic regions (Extended Data Fig. 6c).

**Fig. 6:**
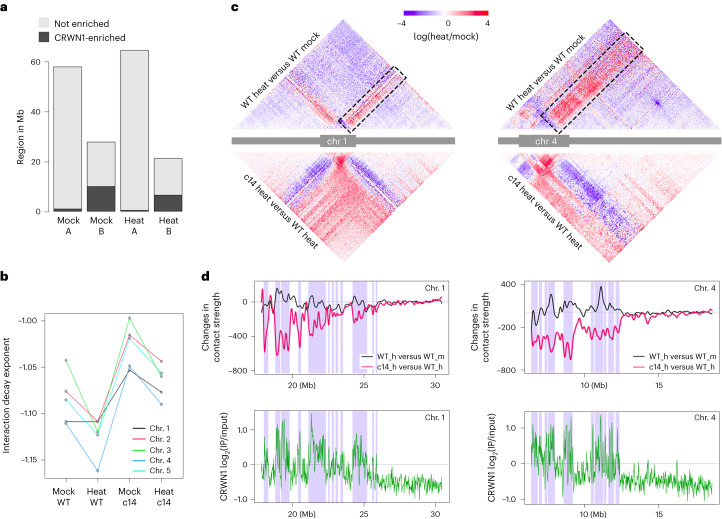
CRWN1 modulates chromatin organization under heat stress. **a**, Distribution of CRWN1-enriched regions among A/B compartments in wild-type plants. For individual samples, chromosome arm regions are grouped according to the A/B compartment annotation. For each group, the regions with and without enrichment by CRWN1 are shown as black and grey blocks, respectively. **b**, IDEs of pericentromeric regions in different samples. IDEs were calculated from normalized Hi-C maps (20 kb) using a distance range of 20 kb to 1 Mb. **c**, Comparison of Hi-C contacts at chromosome 1 (left) and chromosome 4 (right). Colours indicate the difference of chromatin interaction strengths, expressed as the ratio between the two selected samples. The boxes highlight areas describing altered contacts concerning interactions between pericentromeric region and chromosome arm, which are further illustrated in **d**. The pericentromeric region is depicted as a grey block in the chromosome sketch. **d**, CRWN proteins shape chromatin organization under heat stress. Top: difference in chromatin contact between pericentromeric region and chromosome arm; for each curve, positive and negative values indicate stronger and weaker contacts, respectively. Bottom: CRWN1 ChIP–seq signal in heat-stressed plants. The shaded areas depict compartment B annotation in heat-stressed wild-type plants.

**Extended Data Fig. 7:**
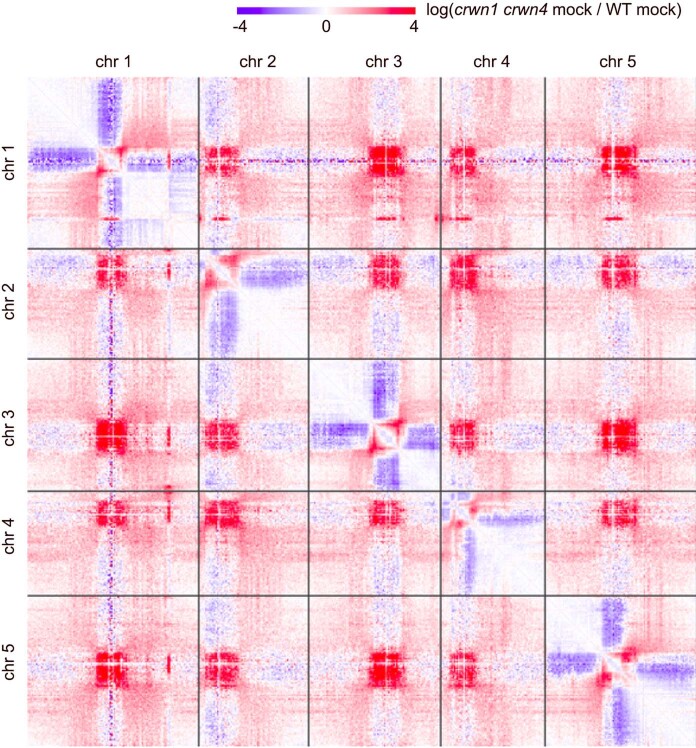
Comparison of genome-wide chromatin interaction patterns between *crwn1 crwn4* and wild-type (WT) plants. Colors indicate the difference of chromatin interaction strengths, expressed as the ratio of the *crwn1 crwn4* mutant over WT sample.

The *crwn1* *crwn4* mutant Hi-C map revealed changes in pericentromeric regions (Extended Data Fig. 7), which is consistent with a recent report showing stronger chromatin contacts within and among individual pericentromeres^21^. Analysis of Hi-C data allows one to recognize chromatin regions that are spatially linked to or separated from each other. In most cases, a binary annotation strategy is applied to group chromatin regions into two spatial compartments^36^. In the nucleus including *Arabidopsis*, these two spatial chromatin compartments (named ‘A/B compartment’ when it was first applied to human Hi-C data) differ in gene expression and epigenetic landscape^35–37^. For chromosome arm regions, we found that neither heat stress nor the loss of CRWNs resulted in drastic changes in the chromatin compartmentalization identity, but they both enhanced chromatin contacts between different spatial compartments (Fig. 5b–d). These changes appeared strongest in heat-stressed *crwn1* *crwn4*. Taking chromosome 1 right arm as an example, among all the plant samples, the Hi-C map of heat-stressed *crwn1* *crwn4* showed the least correlation between spatial compartments (Fig. 5a). Furthermore, CRWN1 displayed preferential enrichment of chromatin binding with the B compartment, and chromatin in heat-stressed *crwn1* *crwn4* showed noticeably the weakest compartmentalization, indicating that CRWNs play a structural role in regulating genome organization under heat stress (Figs. 5d and 6a).

**Extended Data Fig. 8:**
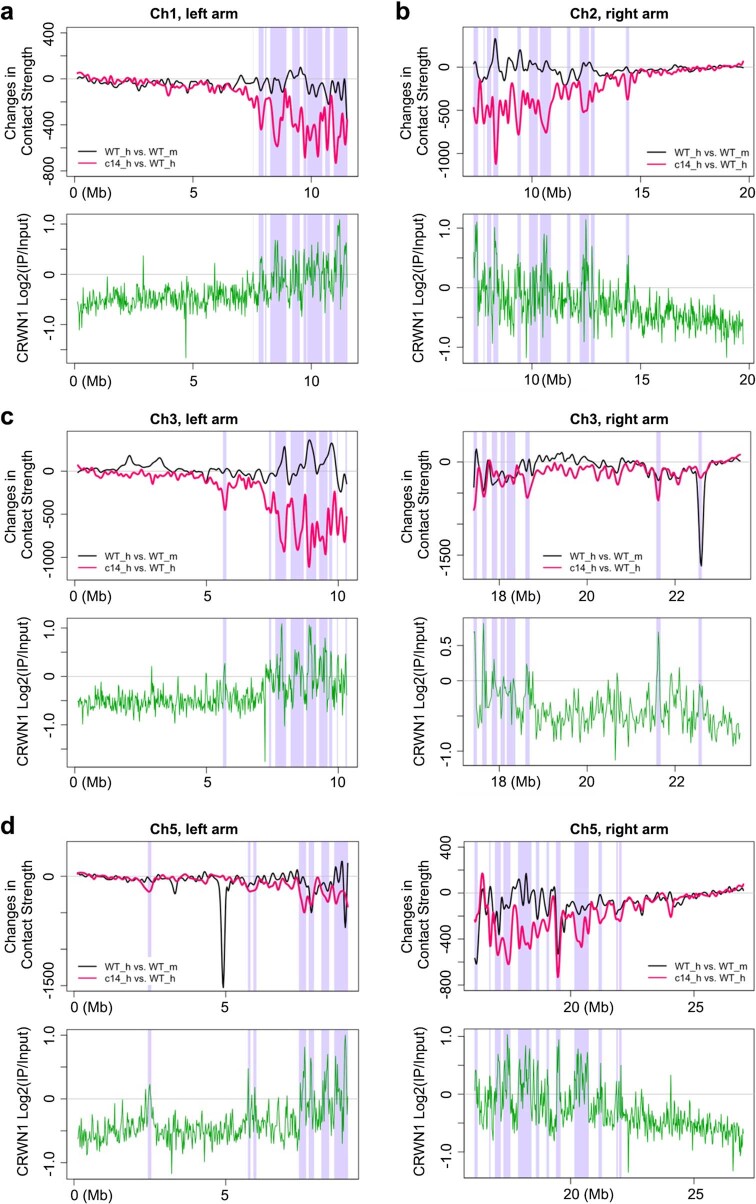
CRWN1 modulates chromatin organization under heat stress. **a–d**, Changes in chromatin contacts between pericentromeric regions and their neighboring chromosome arms. The short arms of chromosomes 2 and 4 are not included. Labels are as in Fig. 6d.

Further support for a structural role of CRWNs in modulating chromatin organization under heat stress was provided by the observation that the heterochromatic pericentromeres acquired more chromatin contacts with chromosome arms under heat stress. Concomitant changes in folding conformation could be reflected by the interaction decay exponents (IDEs) describing chromatin interaction as a function of loci distance (Fig. 6b and Extended Data Fig. 6a)^32^. For each chromosome, heat stress resulted in steeper decrease in chromatin contacts within the pericentromere at long distances, indicating that this stress condition led to heterochromatin decondensation (Fig. 6b). Accordingly, further inspection of Hi-C maps suggested that the decondensed pericentromeres acquired more chromatin contacts with chromosome arms (Fig. 6c, highlighted with dotted-line boxes). Interestingly, the genomic regions that located in chromosome arms and showing stronger chromatin contacts with pericentromeres under heat stress tended to be associated with CRWN1 binding (Fig. 6d, black curves). For *crwn1* *crwn4* under heat stress, compared to wild-type plants, pericentromeres showed decreased chromatin contacts with chromosome arms, which was strongly correlated with CRWN1 ChIP–seq signals (Fig. 6d, red curves, and Extended Data Fig. 8). Taken together, these results indicate that the dynamic CRWN1–chromatin interactions participate in shaping genome organization under heat stress.

## Discussion

In this work, we present data highlighting the dynamic nature of the plant nuclear lamina in response to various stress conditions, such as heat, salt and osmotic stresses (Fig. 2). Since multiple nuclear lamina components interact with each other^14,38^, one would speculate that the assembly of the nuclear lamina relies on the presence of all these proteins. However, the localization of single lamin protein species at the nuclear periphery turned out to be independent of other nuclear lamina components. For example, KAKU4 protein localization remains at the nuclear periphery in *crwn1* *crwn4* double mutant^14^. These results prompted us to speculate that the nuclear localization patterns of lamin proteins are largely independent of each other, and during stress adaptation their disassembly was triggered in parallel. To gain a better understanding on how the plant nuclear lamina responds to stresses, it will be intriguing to further identify which parts or which features of individual lamin proteins enable their dynamic behaviour.

To our surprise, the disassembly of the *Arabidopsis* nuclear lamina under heat stress does not lead to apparent changes in nuclear size and shape, albeit multiple studies have clearly demonstrated that nuclear lamin components, for example, CRWN1, CRWN4 and KAKU4, are indispensable for proper nuclear morphology^7,8,14^. Although we could not verify in our experiments whether the nuclear periphery was free of nuclear lamina under stress, our observation strongly suggests that the localization of tested nuclear lamin components at the nuclear periphery is not necessary to maintain nuclear morphology. Nuclear size in animals is known to be controlled at multiple phases of the cell cycle, including the stage of building the nuclear envelope in newly formed daughter cells (recently reviewed by Cantwell and Dey^39^). Our results imply that CRWNs and KAKU4 proteins play their structural roles concerning nuclear morphology before interphase. During interphase they actively participate in mediating stress responses. In line with this notion, both CRWN1 and CRWN4 assemblies were observed at telophase, when the nuclear envelope reconstruction starts^8^.

The dynamics of the *Arabidopsis* nuclear lamina suggests several future exciting avenues for further research, concerning the underlying regulatory mechanisms, spatial chromatin organization and transcriptional regulation. First, multiple studies on CRWN1 indicate that the plant nuclear lamin component responds to stresses in a highly variable manner. CRWN1 is degraded after infection with virulent bacteria, which is probably mediated by the salicylic acid signalling pathway^16^. On the other hand, it was shown by Sakamoto and colleagues that CRWN1 proteins remained stably localized at the nuclear periphery in roots when plants were stressed with excess copper ions^38^. Here, we reveal that heat-stress treatment causes CRWN1 (as well as other nuclear lamina components CRWN4 and KAKU4) to be redistributed to the nucleoplasm in the absence of protein degradation (Fig. 2). It is unknown how CRWN1 proteins respond differently to these stress stimuli. However, on the basis of the available information on metazoan nuclear lamins^40,41^, which are functionally and structurally comparable (but not with respect to sequence) to the plant counterparts, we speculate that posttranslational modification may confer on plant nuclear lamins the capability to differentiate distinct stress stimuli. Posttranslational modifications of the plant nuclear lamina have not been systematically documented; nonetheless, 10–30 experimentally confirmed phosphorylation sites are reported for CRWN1, CRWN4 and KAKU4 in an *Arabidopsis* phosphoproteomics database^42^. Technically, it is feasible to profile posttranslational modification patterns of a given plant nuclear lamin component under different stress conditions, which could be useful in elucidating how the nuclear lamina reacts differently to diverse stimuli.

Second, plant nuclear lamina components under various stress conditions may interact with diverse genomic regions. In this study, we focused on CRWN1 and found noticeable gain and loss of CRWN1–chromatin interactions during the nuclear lamina disassembly (Fig. 3c), which correlated to changes in gene expression (Fig. 4). Accompanied by changes in spatial distribution, the nuclear lamin proteins may switch to interact with new protein partners. A recent proteomic approach using biotin proximity labelling indicated that KAKU4 interacts with nucleosomes (presumably from chromatin) at the nuclear periphery^18^. Since KAKU4 proteins detached from the nuclear envelope after perceiving stresses (Fig. 2), we speculate that KAKU4 might bind to different genomic loci before and after the nuclear lamina disassembly. In addition, whether CRWN2 and CRWN3, which are homologues of CRWN1 but located throughout the nucleoplasm^8,13^, interact directly with chromatin remains unknown. Nevertheless, we propose that this would be the case since CRWN1 was reported to interact with all its homologues (that is, other CRWNs) in vivo^38^.

Third, given a strong correlation between the localization of chromatin at the plant nuclear periphery and suppressed gene expression^19,20^, it is of great interest to explore to what extent changes in the nuclear lamina at the nuclear periphery are connected to transcriptional regulation in this nuclear compartment. It has been noted that a few genes showed propensity to be repositioned to the nuclear periphery along with transcriptional activation/upregulation, examples of which include copper-associated gene in response to copper toxicity and the *CAB* gene cluster (encoding chlorophyll *a*/*b*-binding proteins) in response to red/far-red light^38,43^. Is such transcriptional regulation associated with changes at the nuclear periphery so that it favours transcription of these genes? Will the disassembly of the plant nuclear lamina convert the nuclear periphery to a place harbouring more active euchromatin? Although such a scenario has not been described in plants, case studies focusing on rod photoreceptor nuclei from nocturnal animals show that withdrawal of lamin–chromatin contacts inverts euchromatin/heterochromatin distribution patterns at the nuclear periphery^44,45^.

## Methods

### Plant materials and growth conditions

Surface-sterilized *Arabidopsis* seeds were sowed on vertical half-strength Murashige and Skoog media plates containing 1% sucrose and 0.3% phytagel. After being stratified at 4 °C for 3 d, the plant materials were grown in a growth chamber (MLR-352-PE from PHCbi) set at 21 °C and long day (16 h light/8 h dark) conditions. Mutants used in this study were *crwn1-1* (SALK_025347), *crwn4-1* (SALK_079296) and *kaku4-2* (SALK_076754), which were ordered from the Nottingham *Arabidopsis* Stock Centre.

For heat-stress treatment, 11-day-old plants were transferred to another growth chamber of the same model and with identical settings, except that the temperature was set at 37 °C when lights were switched on. Plants were collected three days later for analyses. For salt and osmotic stress treatment, 12-day-old plants were transferred to half-strength Murashige and Skoog plates supplemented with 150 mM sodium chloride or 5% PEG 8000, respectively. Plants were collected two days later for analyses.

Plant nuclei were extracted essentially as described in our earlier work^46^. The subsequent nuclei sorting was performed with a S3e Cell Sorter (Bio-Rad) according to our established protocol^10^. The extracted nuclei were stained with 0.5 µM DAPI to reveal their ploidy levels; if not otherwise specified, only 2C nuclei were collected for downstream experiments.

### Plasmid construction

For the *CRWN4:2HA* construct, a tandem HA tag (2HA) was inserted after the 850th amino acid residue of the CRWN4 protein, generating a *CRWN4:2HA* fusing construct that fully rescued *crwn4* loss-of-function mutant phenotypes. The two fragments of this *CRWN4:2HA* construct were amplified with primers: 5′-ACTAATCTTTTCTACTAGCTTAAC-3′ in combination with 5′-AGGGTATCCAGCATAATCTGGTACGTCGTATGGGTATCCAGTACATCGTTTTATCCATGA-3′; and 5′-GATTATGCTGGATACCCTTACGACGTACCAGATTACGCTAATCTGATTTTCAAGACTTCTCCA-3′ in combination with 5′-GCTACGAGCTACTTCGATGATAC-3′, respectively. These two fragments, which collectively cover the genomic fragment of the *CRWN4* locus and its 2 kb promoter regions, were assembled with overlapping PCR and subsequently amplified with primers 5′-ACTAATCTTTTCTACTAGCTTAAC-3′ and 5′-GCTACGAGCTACTTCGATGATAC-3′. The PCR product was cloned into the *pFK206* vector^47^.

For the *KAKU4:GFP* construct, the genomic region containing 2 kb upstream of *KAKU4* and its coding sequence was amplified with oligonucleotides 5′-GCATAGAACGAGGAATACAGG-3′ and 5′-CTGCCTCCTGCAGCTCCGGATTTGGCCCGTCCTTTGCCTC-3′, and the GFP complementary DNA was amplified with oligonucleotides 5′-TCCGGAGCTGCAGGAGGCAGCGCGGCCGCTGTGAGCAAGGG-3′ and 5′-TTATCCGGACTTGTACAGCTCG-3′. These two PCR fragments were purified and cloned into the *pFK206* vector with a Gibson assembly reaction, by which the *GFP* sequence was appended to the C terminus of *KAKU4*.

### FISH and immunohistostaining

Bacterial artificial chromosome (BAC) probes were generated by the nick translation DNA labelling system (Roche, catalogue no. 11745808910). The green and red probes described in this study were labelled by digoxigenin (DIG) and 2,4-dinitrophenyl (DNP), respectively. The labelled BACs were pooled in a hybridization mix (50% formamide, 10% dextran sulfate, 2× SSC, 50 mM sodium phosphate (pH 7.0)). The working concentration of each labelled BAC was 1 ng μl^−1^. Individual BACs are listed in Supplementary Table 1.

FISH experiments were performed according to ref. ^48^. Briefly, around 5,000 nuclei in 20 μl PBS buffer were incubated at 65 °C for 30 min. The nuclei were subsequently mixed with 10 μl of 0.1 mg ml^−1^ RNase A and spread into a circle drawn on a glass slide using an ImmEdge pen. After incubating the slides at 37 °C in a hybridizer (ThermoBrite, model 07J91-020) for 1 h, the slides were dipped up and down for 1 min each in a graded ethanol series (30%, 60%, 80%, 90%, 95%, 100% EtOH) for dehydration. For immunohistostaining, an antigen retrieval step was performed by incubating the slides in boiling solution (10 mM sodium citrate at pH 6.0) for 12 min in a microwave oven at 700 W. After antigen retrieval, slides were postfixed in 4% formaldehyde solution for 10 min, followed by dehydration in a graded ethanol series and then air-dried. The treated slides were used for FISH or immunohistostaining experiments. For FISH experiments, the probe hybridization, slide washing and signal detection steps were performed according to our published protocol with minor changes^19^. DIG-labelled probes were detected with 1:10 diluted DIG Alexa Fluor 488-conjugated mouse antibody (Biotechne, catalogue no. IC7520G), and DNP-labelled probes were detected with 1:500 diluted DNP rabbit antibody (ThermoFisher, catalogue no. 04-8300) and 1:150 diluted anti-rabbit Alexa Fluor 546-conjugated goat antibody (ThermoFisher, catalogue no. A-11035). For immunohistostaining, the HA-tagged protein of interest was detected with 1:500 diluted HA tag Alexa Fluor 647 conjugated mouse antibody (ThermoFisher, catalogue no. 26183-A647). After antibody incubation, the slides were washed with 4× SSC 0.2% Tween 20 in a foil-wrapped jar at room temperature, three times for 5 min each. Finally, slides were mounted with 5 μl SlowFade Diamond Antifade Mountant (Invitrogen, catalogue no. S36964).

### Microscopy and image processing

Confocal images were captured with a Zeiss LSM 700 system. For FISH experiments, a single image was taken from the central focal plane of individual nuclei. Image analyses were performed with ImageJ^49^. The distance between a FISH signal spot and the nuclear periphery was approximated as the distance between its estimated barycentre and the edge of the DAPI staining. We noticed that a fraction of the observed nuclei showed split or scattered FISH signal patterns, making it difficult to estimate the distance between the probed genomic region to the nuclear envelope (Supplementary Fig. 1). These nuclei were excluded from the analysis. During FISH image acquisition, we also excluded those nuclei in which FISH signals were apparently located close to the top or the bottom of nuclei, because they would largely mislead distance calculations. These nuclei could be easily recognized by moving the sample stage along the *z* axis to reveal that the focal plane containing FISH signals could only capture DAPI signals with a smaller area (Supplementary Fig. 5a). For immunostaining, an image of the central focal plane of a nucleus was taken to analyse the distribution of the protein of interest. Images of chromosome painting were acquired with a Zeiss LSM 880 system. Quantification of nuclear size was performed with images of DAPI-stained nuclei taken with an Olympus IX83 fluorescence microscope. The Olympus cellSens software (v.3.10.12201.0) was used to measure the area of nuclei in the images, which was used as an approximation to nuclear size.

### Protein extraction and western blot

Protein extraction from aerial tissues was performed with sample homogenization using protein extraction buffer (50 mM Tris–HCl, 150 mM NaCl, 0.1% Tween 20) supplemented with 1% β-mercaptoethanol, 0.1 M PMSF and protease inhibitor (protease inhibitor cocktail tablets; Roche). The homogenate was centrifuged for 10 min at 4 °C at 13,300*g*. The supernatant was recovered and analysed by western blot. The following antibodies were used to detect protein of interest: for HA-tagged proteins, anti-HA-HRP (Santa Cruz Biotechnology, catalogue no. sc-7392); for GFP-tagged proteins, anti-GFP (Abcam, catalogue no. ab290) followed by anti-rabbit HRP conjugate (Sigma-Aldrich, catalogue no. A6154). All of the antibodies were used with 1:5,000 dilution. After chemiluminescence detection, membranes were stained with Coomassie blue.

### Chromatin immunoprecipitation and library sequencing

*Arabidopsis* shoots were fixed under vacuum for 30 min with 1% formaldehyde in MC buffer (10 mM potassium phosphate, pH 7.0, 50 mM NaCl, 0.1 M sucrose) at room temperature. Fixation was terminated by replacing the solution with 0.15 M glycine in MC buffer under vacuum for 10 min at room temperature. Approximately 1 g of fixed tissue was homogenized and resuspended in nuclei isolation buffer (20 mM HEPES, pH 8.0, 250 mM sucrose, 1 mM MgCl_2_, 5 mM KCl, 40% glycerol, 0.25% Triton X-100, 0.1 mM PMSF, 0.1% 2-mercaptoethanol) and filtered with double-layered miracloth (Millipore). Isolated nuclei were resuspended in 0.5 ml sonication buffer (10 mM potassium phosphate, pH 7.0, 0.1 mM NaCl, 0.5% sarkosyl, 10 mM EDTA), and chromatin was sheared by sonication with a QSONICA sonicator Q800R3 to achieve average fragment size around 400 base pairs (bp). Next, 50 µl 10% Triton X-100 was mixed with the sonicated sample, and 25 µl of the mixture was saved as input sample. The rest of the sheared chromatin was mixed with an equal volume of immunoprecipitation (IP) buffer (50 mM HEPES, pH 7.5, 150 mM NaCl, 5 mM MgCl_2_, 10 µM ZnSO_4_, 1% Triton X-100, 0.05% SDS) and incubated with Pierce anti-HA magnetic beads (Thermo Fisher) at 4 °C for 2 h. The beads were washed at 4 °C as follows: 2× with IP buffer, 1× with IP buffer having 500 mM NaCl and 1× with LiCl buffer (0.25 M LiCl, 1% NP-40, 1% deoxycholate, 1 mM EDTA, 10 mM Tris–HCl pH 8.0) for 3 min each. After a brief wash with TE buffer (10 mM Tris–HCl pH 8.0, 1 mM EDTA), the beads were resuspended in 200 µl elution buffer (50 mM Tris–HCl, pH 8.0, 200 mM NaCl, 1% SDS, 10 mM EDTA) at 65 °C for 6 h, followed by proteinase K treatment at 45 °C for 1 h. DNA was purified with a MinElute PCR purification kit (Qiagen), and then used for qPCR or converted into sequencing libraries following the NEBNext Ultra II DNA Library Prep Kit (NEB). After sequencing, ChIP–seq reads were mapped to the TAIR10 genome with Bowtie 2 (v.2.2.4). Subsequently, ChIP–seq peak calling was done with MACS2 v.2.1.1 (ref. ^50^); the reads from input were used as control. Detailed description of the parameters used for peak calling and the results can be found in Supplementary Data 1. For ChIP–qPCR, the relative enrichment of tested loci was normalized to the *TUB2* locus, which does not belong to PLADs and is not bound by CRWN1. Oligonucleotides used for ChIP–qPCR are listed in Supplementary Table 2.

### Gene expression analyses

RNA sequencing (RNA-seq) was performed with two biological replicates per sample. Total RNA was extracted from aerial parts of seedlings using a RNeasy Plant Mini Kit (Qiagen). RNA-seq library preparation was performed as previously described^10^. RNA-seq sequencing reads were aligned against the Araport11 annotation using TopHat2 (v.2.1.1) with default parameters, and were further assigned to genes using the R package GenomicAlignments^51–53^. Differentially expressed genes were identified with the R package DESeq2 (ref. ^54^). We used criteria of false discovery rate smaller than 0.01 and log_2_(expression fold change) more than 1.6 to call upregulated and downregulated genes. Details of the reads count table, gene expression measurement (in reads per kilobase per million mapped reads) and differentially expressed genes can be found in Supplementary Data 2.

For individual gene expression analysis, total RNA was isolated from samples using the RNeasy Plant Mini Kit (Qiagen) following the manufacturer’s instructions. The RNA was then treated with DNase I (Thermo Scientific) to remove any contaminating DNA. Subsequently, the RNA was reverse transcribed to cDNA using SuperScript II Reverse Transcriptase (Invitrogen). Quantitative RT–PCR was conducted on a CFX96 real-time system (Bio-Rad) using qPCRBIO SyGreen Mix ROX (Lo-ROX) (PCR Biosystems) with gene-specific primers listed in Supplementary Table 2.

### In situ Hi-C

In situ Hi-C libraries were prepared essentially as previously described^55^. In total, two Hi-C library replicates for each sample were made, and for each replicate around 0.5 g of fixed sample was homogenized for nuclei isolation. Nuclei were resuspended with 150 µl 0.5% SDS and split into three tubes. After penetration at 62 °C for 5 min, SDS was quenched by adding 145 µl water and 25 µl 10% Triton X-100, and incubated at 37 °C for 15 min. Subsequently, chromatin was digested overnight at 37 °C with 50 U DpnII (NEB) in each tube. The next day, DpnII was inactivated by incubating at 62 °C for 20 min. Then, sticky ends were filled in by adding 1 µl of 10 mM dTTP, 1 µl of 10 mM dATP, 1 µl of 10 mM dGTP, 10 µl of 1 mM biotin-14-dCTP, 29 µl water and 40 U Klenow fragment (Thermo Fisher), and incubated at 37 °C for 2 h. After adding 663 µl water, 120 µl 10× blunt-end ligation buffer (300 mM Tris–HCl, 100 mM MgCl_2_, 100 mM DTT, 1 mM ATP, pH 7.8) and 40 U T4 DNA ligase (Thermo Fisher), proximity ligation was carried out at room temperature for 4 h. Then, three tubes of ligation products were centrifuged, and nuclei pellets were resuspended and combined with 650 µl SDS buffer (50 mM Tris–HCl, 1% SDS, 10 mM EDTA, pH 8.0). After treatment with 10 µl proteinase K (Thermo Fisher) at 55 °C for 30 min, de-cross-linking was performed by adding 30 μl 5 M NaCl and incubating at 65 °C overnight. DNA was recovered and subsequently treated with RNase A at 37 °C for 30 min. After purification, 3–5 μg Hi-C DNA was topped to 130 μl with TE buffer (10 mM Tris–HCl, 1 mM EDTA, pH 8.0) and sheared with a Q800R3 sonicator (QSONICA) by using the following setting: 25% amplitude, 15 s ON, 15 s OFF, pulse-on time for 4.5 min, to achieve fragment size shorter than 500 bp. Sonicated DNA was purified with Ampure beads to recover fragments longer than 300 bp. Then, with a 50 μl reaction volume, the DNA was mixed with 0.5 μl 10 mM dTTP, 0.5 μl 10 mM dATP and 5 U T4 DNA polymerase and incubated at 20 °C for 30 min to remove biotin from unligated DNA ends. After that, the DNA was purified with Ampure beads, and continued with end repair and adaptor ligation using the NEBNext Ultra II DNA Library Prep Kit (NEB). Ligated DNA was affinity purified with Dynabeads MyOne Streptavidin C1 beads (Invitrogen) as described^56^, and further amplified with 12 PCR cycles. The libraries were sequenced on an Illumina Novaseq instrument with 2 × 150 bp reads.

Reads mapping to the TAIR10 genome with Bowtie 2 (v.2.2.4), removal of PCR duplicates and reads filtering were performed as previously described^56^. Hi-C reads of each sample are summarized in Supplementary Data 3. Hi-C map normalization was performed by using an iterative matrix correction function in the ‘HiTC’ package in the R program^57^. For all Hi-C maps, the iterative normalization process was stopped when the eps value, which reflected how similar the matrices in two consecutive correction steps were, dropped below 1 × 10^−4^. The bin size setting for genome-wide Hi-C maps was 20 kb. In addition, the filtered Hi-C reads were used to create hic files with the juicer tool for interactive Hi-C map inspection^58^. The annotation of A/B compartment of chromosome arms is available in Supplementary Data 3. Genomic coordinates of individual pericentromeric regions for computing the IDE were defined as follows: Chr. 1: 11.5–17.7 Mb; Chr. 2: 1.1–7.2 Mb; Chr. 3: 10.3–17.3 Mb; Chr. 4: 1.5–6.3 Mb; Chr. 5: 9.0–16.0 Mb (ref. ^59^).

### Reporting summary

Further information on research design is available in the Nature Portfolio Reporting Summary linked to this article.

## Supplementary information

Supplementary Information Supplementary Figs. 1–5.

Reporting Summary

Supplementary Tables 1 and 2 1: BACs used for pairwise FISH comparison. 2: Sequences of oligonucleotides used for quantitative PCR.

Supplementary Data 1 Results of ChIP–seq peak calling. The last datasheet in this file (that is, ‘CRWN1-targets’) describes the classification of genes based on their enrichment by CRWN1 under different growth conditions.

Supplementary Data 2 Gene expression quantification and DEG calling.

Supplementary Data 3 Overview of Hi-C datasets and AB compartment annotation.

Supplementary Data 4 Statistical source data for supplementary figures.

## Data Availability

Short read data of in situ Hi-C, ChIP–seq and RNA-seq are publicly available at NCBI Sequence Read Archive under accession number PRJNA870030. Large datasets, such as normalized Hi-C matrices and BigWig ChIP–seq track files are available in the figshare repository, which are accessible at 10.6084/m9.figshare.21370560.v1. Source data are provided with this paper.

## Source data

Source Data Fig. 1 Statistical source data.
Source Data Fig. 2 Western blots and membrane staining.
Source Data Fig. 4 Statistical source data.
Source Data Fig. 5 Statistical source data.
Source Data Extended Data Fig. 4 Statistical source data.

## Code availability

All scripts used in this study are available upon request.
