# Supplementary material for: The plant nuclear lamina disassembles to regulate genome folding in stress conditions
Source: Nat Plants. 2023 Jul 3;9(7):1081–93. doi: 10.1038/s41477-023-01457-2 (PMC10356608; doi:10.1038/s41477-023-01457-2)
Supplement: Supplementary file 1 — Supplementary Figs. 1–5. [file 41477_2023_1457_MOESM1_ESM.pdf]

nature plants

Article

https://doi.org/10.1038/s41477-023-01457-2

The plant nuclear lamina disassembles to regulate genome folding in stress conditions

In the format provided by the authors and unedited

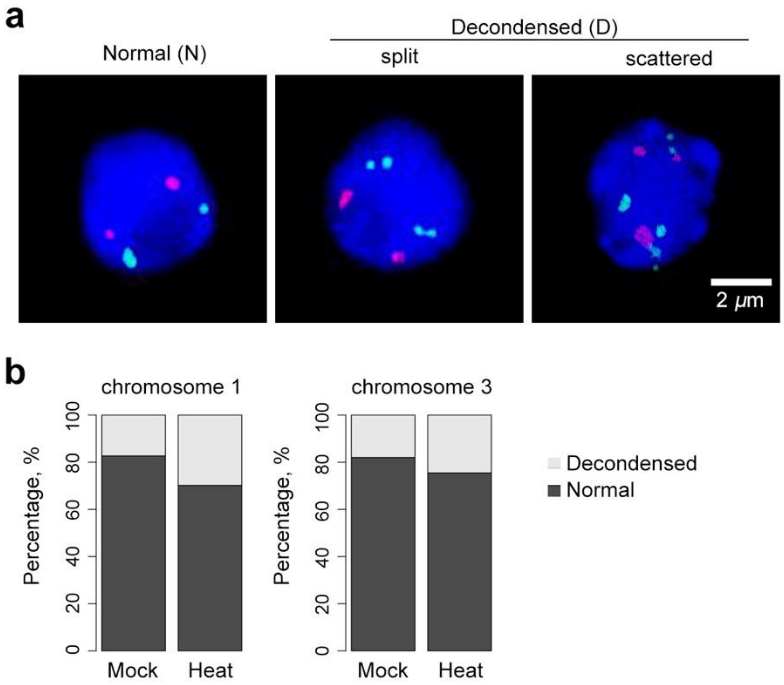

Supplementary Figure 1. Categorization of FISH patterns in nuclei isolated from mock- and heat stress-treated plants. a, Representative images of PLAD (green) and non-PLAD (red) probes. FISH signals indicating decondensed genomic regions were not included in distance measurement, which is shown in Fig. 1c. Images are representatives from two independent experiments with similar patterns. **b,** Percentage of individual categories.

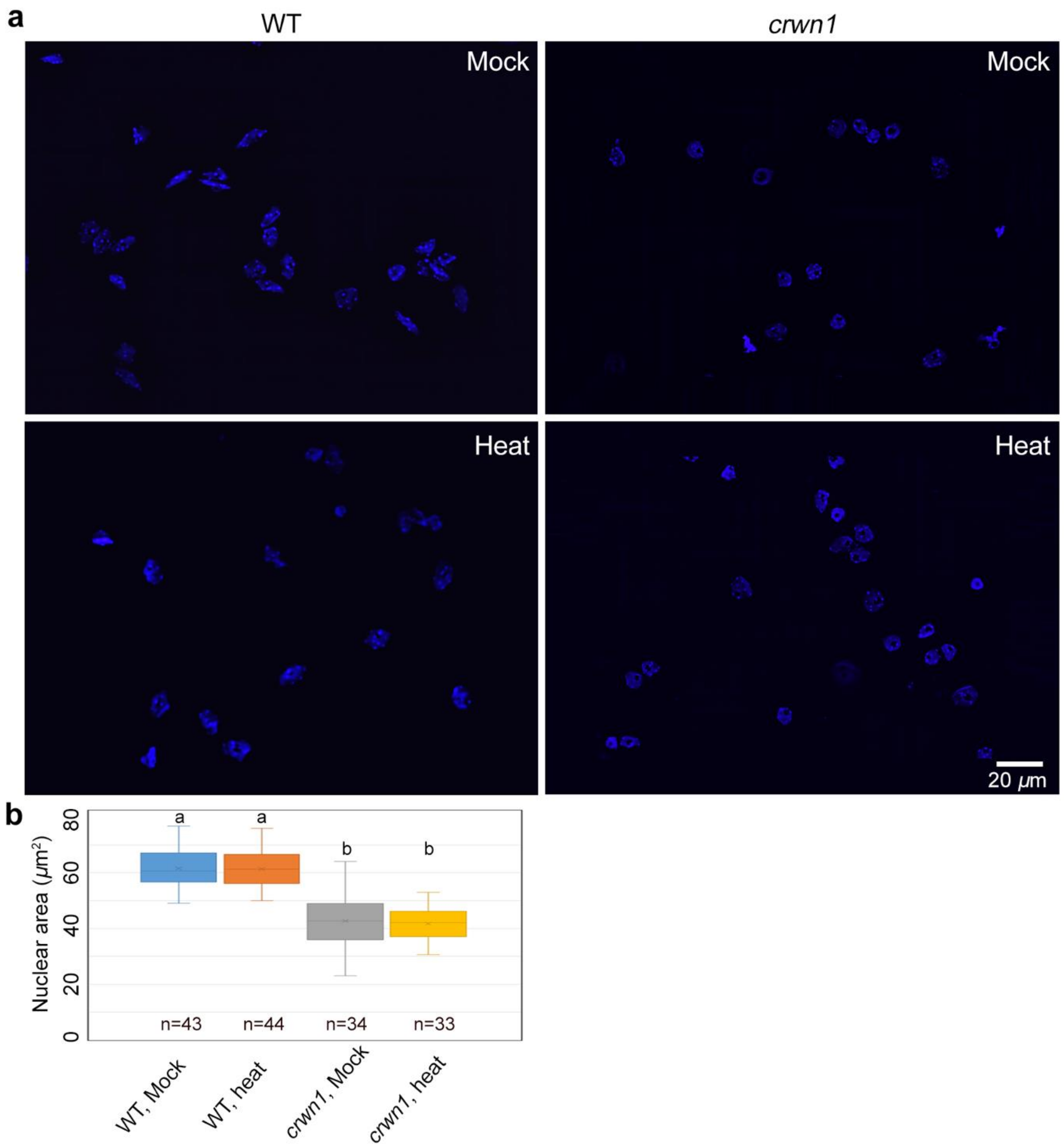

Supplementary Figure 2. Heat stress does not influence nuclear size. a, Comparison of 8C nuclei isolated from heat-stressed and control plant leaves. Images are representatives from at least two independent experiments with similar patterns. **b,** Comparison of nuclear size, which is approximated as the area occupied by a nucleus in the microscope image. The box plots indicate the median (line within the box), the lower and upper quartiles (box), margined by the largest and smallest data points that are still within the interval of 1.5 times the interquartile range from the box (whiskers); outliers are not shown. Boxplots sharing the same alphabet labels on top are not significantly different from each other, determined by one-way ANOVA with Tukey's multiple comparison test ($p > 0.05$).

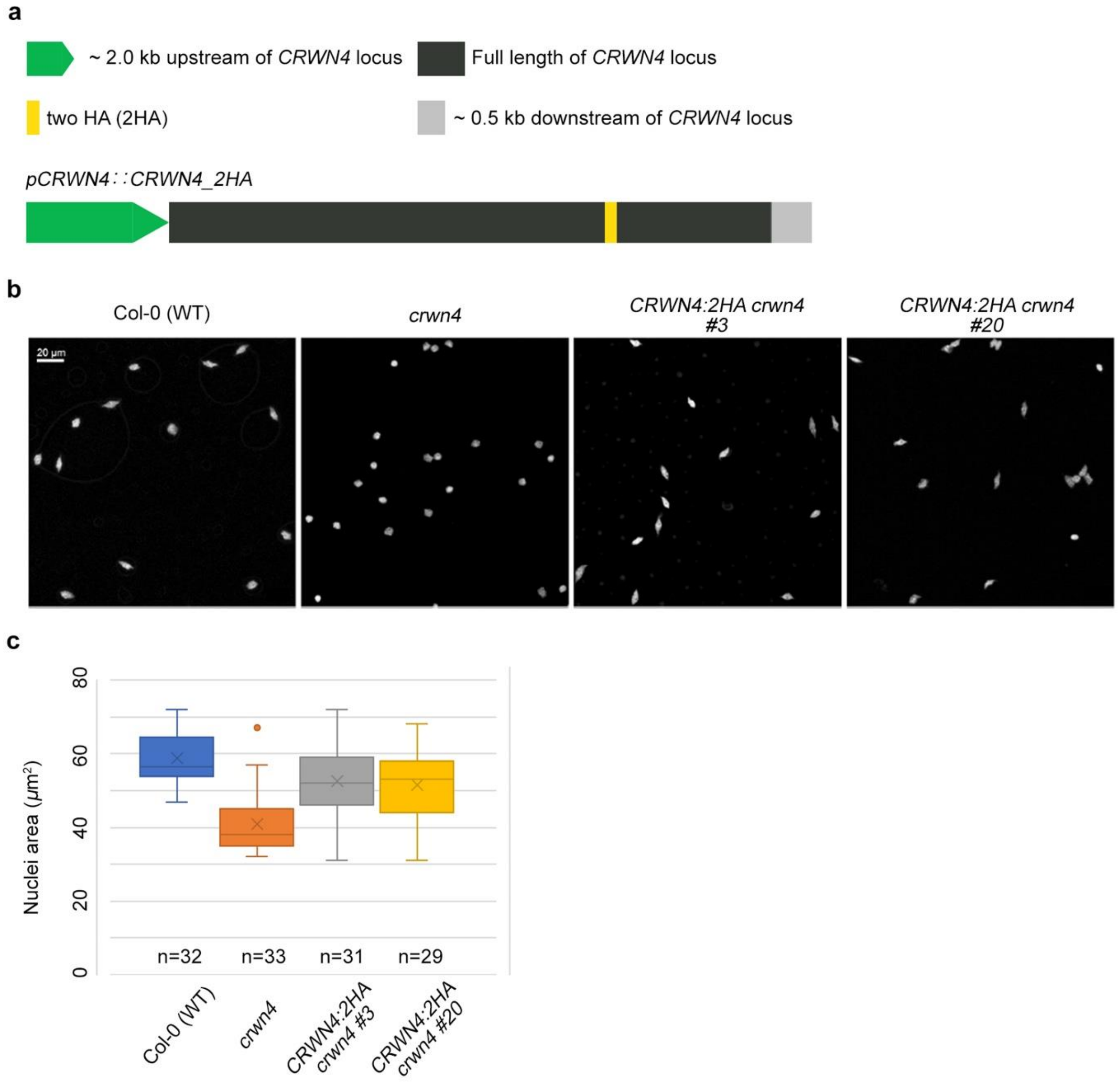

Supplementary Figure 3. Functional complementation of *crwn4* phenotypes by the *CRWN4:2HA* transgene. a, A sketch showing the *pCRWN4:CRWN4:2HA* transgene. **b,** Comparison of nuclear morphology of 8C nuclei of wild-type, *crwn4*, and two lines of *pCRWN4:CRWN4:2HA* plants. Images are representatives from two independent experiments with similar patterns. **c,** Boxplots of nuclear sizes of 8C nuclei isolated from different plants. The box plots indicate the median (line within the box), the lower and upper quartiles (box), margined by the largest and smallest data points that are still within the interval of 1.5 times the interquartile range from the box (whiskers).

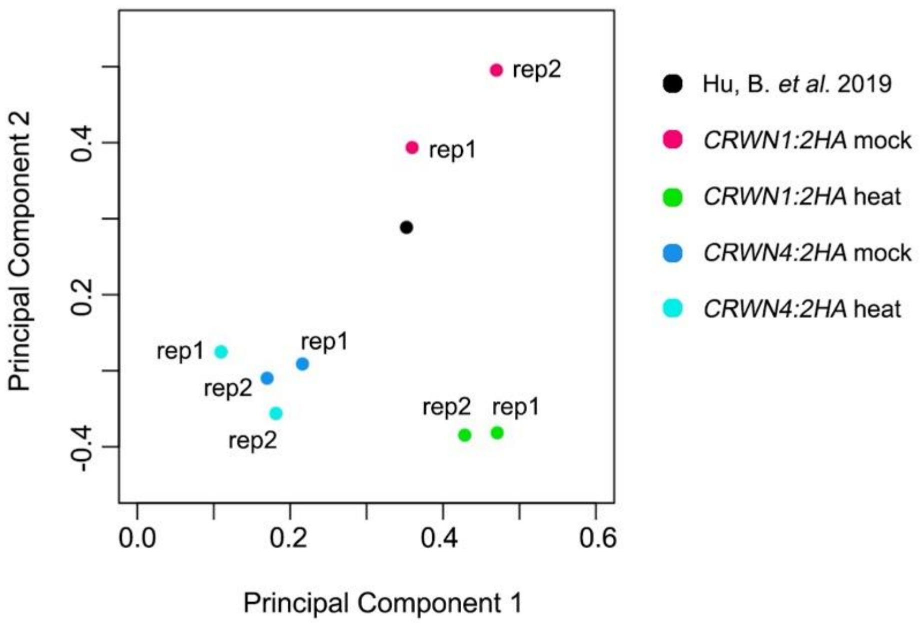

Supplementary Figure 4. Principal component analysis of ChIP-seq samples. Genome-wide ChIP-seq signals (normalized against input) at 20 kb window size are compared. The black dot indicates a previously published CRWN1:2HA ChIP-seq data derived from 10-day-old seedlings [1].

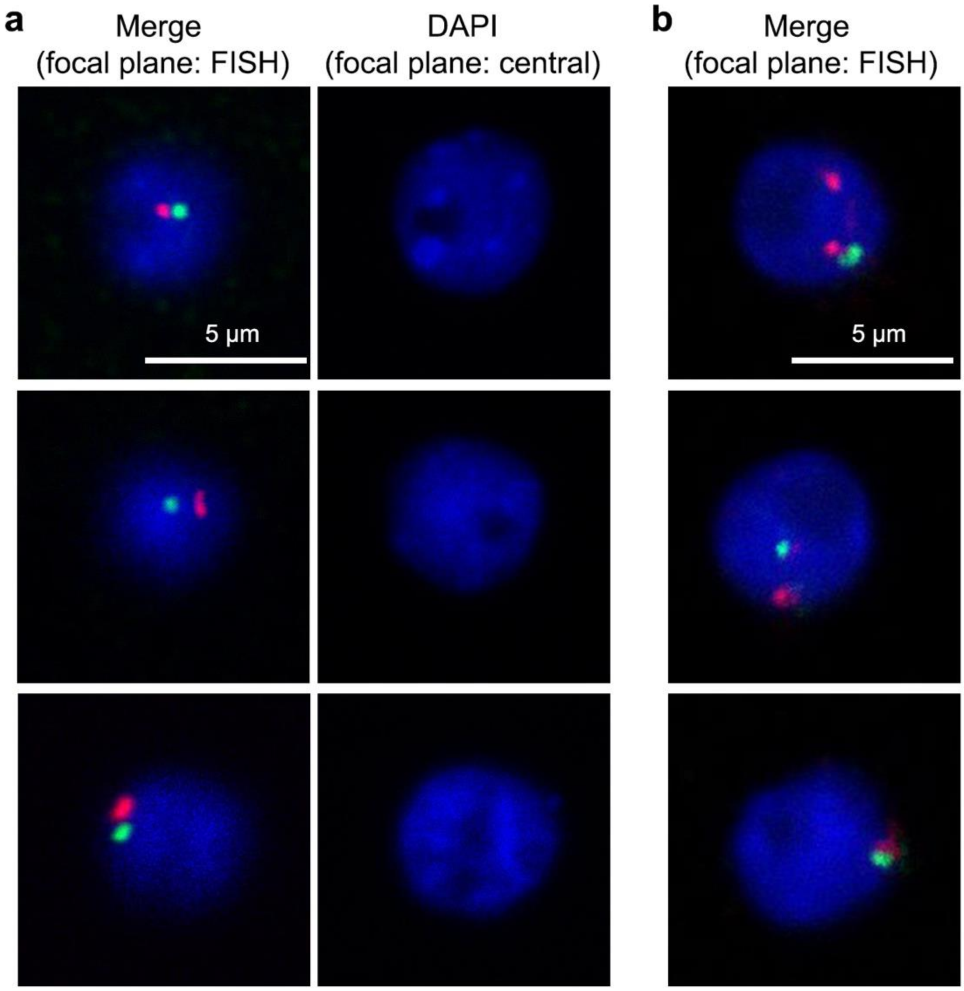

Supplementary Figure 5. Excluding outliers from FISH data analysis. a, Examples of three nuclei in which the FISH signals are close to the top or the bottom of the nuclei. For each nucleus, the panel shown on the right-hand side is the DAPI image taken from the central focal plane. **b,** Examples of nuclei with FISH signals suitable for further analysis. Images in **a** and **b** are representatives from at least two independent experiments with similar patterns.

References

1. Hu, B. *et al.* Plant lamin-like proteins mediate chromatin tethering at the nuclear periphery. *Genome Biol* **20**, 87 (2019).
