# Supplementary material for: The plant nuclear lamina disassembles to regulate genome folding in stress conditions
Source: Nat Plants. 2023 Jul 3;9(7):1081–93. doi: 10.1038/s41477-023-01457-2 (PMC10356608; doi:10.1038/s41477-023-01457-2)
Supplement: Supplementary file 2 — Reporting Summary [file 41477_2023_1457_MOESM2_ESM.pdf]

nature portfolio

Corresponding author(s): Chang Liu

Last updated by author(s): 30.05.2023

Reporting Summary

Nature Portfolio wishes to improve the reproducibility of the work that we publish. This form provides structure for consistency and transparency in reporting. For further information on Nature Portfolio policies, see our Editorial Policies and the Editorial Policy Checklist.

Statistics

For all statistical analyses, confirm that the following items are present in the figure legend, table legend, main text, or Methods section.

n/a	Confirmed	
☐	☒	The exact sample size (*n*) for each experimental group/condition, given as a discrete number and unit of measurement
☐	☒	A statement on whether measurements were taken from distinct samples or whether the same sample was measured repeatedly
☐	☒	The statistical test(s) used AND whether they are one- or two-sided<br>*Only common tests should be described solely by name; describe more complex techniques in the Methods section.*
☒	☐	A description of all covariates tested
☒	☐	A description of any assumptions or corrections, such as tests of normality and adjustment for multiple comparisons
☐	☒	A full description of the statistical parameters including central tendency (e.g. means) or other basic estimates (e.g. regression coefficient) AND variation (e.g. standard deviation) or associated estimates of uncertainty (e.g. confidence intervals)
☐	☒	For null hypothesis testing, the test statistic (e.g. *F*, *t*, *r*) with confidence intervals, effect sizes, degrees of freedom and *P* value noted<br>*Give P values as exact values whenever suitable.*
☒	☐	For Bayesian analysis, information on the choice of priors and Markov chain Monte Carlo settings
☒	☐	For hierarchical and complex designs, identification of the appropriate level for tests and full reporting of outcomes
☐	☒	Estimates of effect sizes (e.g. Cohen's *d*, Pearson's *r*), indicating how they were calculated

Our web collection on statistics for biologists contains articles on many of the points above.

Software and code

Policy information about availability of computer code

Data collection	All sequencing samples generated in this study were prepared in house and sequenced on Illumina platforms.
Data analysis	Bowtie 2 (v2.2.4)<br>MACS2 (v2.1.1.20160309)<br>TopHat 2 (v2.1.1)<br>R (v4.1.0)<br>Olympus cellSens (v3.10.12201.0)<br>Detailed parameters are described in relative sections in "Methods".

For manuscripts utilizing custom algorithms or software that are central to the research but not yet described in published literature, software must be made available to editors and reviewers. We strongly encourage code deposition in a community repository (e.g. GitHub). See the Nature Portfolio guidelines for submitting code & software for further information.

Data

Policy information about availability of data

All manuscripts must include a data availability statement. This statement should provide the following information, where applicable:

- Accession codes, unique identifiers, or web links for publicly available datasets
- A description of any restrictions on data availability
- For clinical datasets or third party data, please ensure that the statement adheres to our policy

Short read data of in situ Hi-C, ChIP-seq, and RNA-seq are publicly available at NCBI Sequence Read Archive under accession number PRJNA870030.
Large datasets, such as Hi-C matrices and ChIP-seq track files in 100 bp bin size are available in the figshare repository, which are accessible with the following link: https://figshare.com/s/8dc4d77ca579b73bbbe4 with Digital Object Identifier (DOI) 10.6084/m9.figshare.21370560.

Human research participants

Policy information about studies involving human research participants and Sex and Gender in Research.

Reporting on sex and gender	not applicable
Population characteristics	not applicable
Recruitment	not applicable
Ethics oversight	not applicable

Note that full information on the approval of the study protocol must also be provided in the manuscript.

Field-specific reporting

Please select the one below that is the best fit for your research. If you are not sure, read the appropriate sections before making your selection.

☒ Life sciences ☐ Behavioural & social sciences ☐ Ecological, evolutionary & environmental sciences

For a reference copy of the document with all sections, see nature.com/documents/nr-reporting-summary-flat.pdf

Life sciences study design

All studies must disclose on these points even when the disclosure is negative.

Sample size	No sample-size calculation was performed for each replicate. For ChIP, gene expression, FISH, and Hi-C experiments, the weight of each batch of harvested samples was approximately 0.5g, consisting of at least 50 seedlings, which was required as the starting material. For these experiments, such a sample size is widely accepted by the Arabidopsis research community.<br>Sample sizes of all box plots in this study were intrinsically linked to samples fulfilling a given selection criterion, which was described clearly in the manuscript.
Data exclusions	No data was excluded from the analyses, except for genomic regions with poor mappability in Hi-C analysis.
Replication	ChIP-qPCR 3 biological replicates; ChIP-seq data: 2 biological replicates; RNA-seq data: 2 biological replicates; RT-qPCR: 3 biological replicates; Hi-C: 2 biological replicates. Immunostaining and FISH experiments: 3 biological replicates. All the attempts at replication were successful. Every two consecutive replications were at least separated by two weeks.
Randomization	Plants with identical sample identity were grown in at least three pots or on three half-strength MS medium plates, which were placed randomly in the growth chamber.<br>For sample harvesting, randomization was also applied, in which control- and heat-stressed plants of the same genotype were randomly chosen from the corresponding pots or medium plates.
Blinding	Blinding was not applicable. For both the experiments in wet lab and NGS data analyses, we applied identical protocol and pipeline to individual samples, respectively.

Reporting for specific materials, systems and methods

We require information from authors about some types of materials, experimental systems and methods used in many studies. Here, indicate whether each material, system or method listed is relevant to your study. If you are not sure if a list item applies to your research, read the appropriate section before selecting a response.

Materials & experimental systems

n/a	Involved in the study
☐	☒ Antibodies
☒	☐ Eukaryotic cell lines
☒	☐ Palaeontology and archaeology
☒	☐ Animals and other organisms
☒	☐ Clinical data
☒	☐ Dual use research of concern

Methods

n/a	Involved in the study
☐	☒ ChIP-seq
☒	☐ Flow cytometry
☒	☐ MRI-based neuroimaging

Antibodies

Antibodies used

The following commercial antibodies were used: anti-HA-HRP (sc-7392, Santa Cruz Biotechnology); anti-GFP (ab290, Abcam) ;anti-rabbit HRP conjugate (A6154, Sigma-Aldrich); ; Anti-rabbit Alexa Fluor 546-conjugated goat antibody (ThermoFisher, Catalog no. A-11035); anti-DNP rabbit antibody (ThermoFisher, Catalog no. 04-8300); anti-DIG Alexa Fluor 488-conjugated mouse antibody (Biotechne, Catalog no. IC7520G); anti-HA Alexa Fluor 647 conjugated mouse antibody (ThermoFisher, Catalog no. 26183-A647).

Validation

All antibodies have been validated by the vendors. Primary antibodies that recognize HA- or GFP-tagged proteins were also validated by ourselves by including wild-type materials (for western blot and immunohistostaining experiments) as negative controls. Additional antibodies validation for FISH experiments were performed by removing probes from the hybridization step.

Addition information from manufacturers:
anti-HA beads:
https://www.thermofisher.com/order/catalog/product/de/en/88836

anti-HA-HRP, sc-7392:
https://www.scbt.com/p/ha-probe-antibody-f-7

anti-GFP, ab290:
https://www.abcam.com/products/primary-antibodies/gfp-antibody-ab290.html

anti-rabbit HRP conjugate, A6154:
https://www.sigmaaldrich.com/DE/de/product/sigma/a6154

Anti-rabbit Alexa Fluor 546-conjugated goat antibody, A-11035:
https://www.thermofisher.com/antibody/product/Goat-anti-Rabbit-IgG-H-L-Highly-Cross-Adsorbed-Secondary-Antibody-Polyclonal/A-11035

anti-DNP rabbit antibody, 04-8300:
https://www.thermofisher.com/antibody/product/DNP-Antibody-clone-LO-DNP-2-Monoclonal/04-8300

anti-DIG Alexa Fluor 488-conjugated mouse antibody: IC7520G:
https://www.rndsystems.com/products/digoxigenin-alexa-fluor-488-conjugated-antibody-611621_ic7520g

anti-HA Alexa Fluor 647 conjugated mouse antibody, 26183-A647:
https://www.thermofisher.com/antibody/product/HA-Tag-Antibody-clone-2-2-2-14-Monoclonal/26183-A647

ChIP-seq

Data deposition

☒ Confirm that both raw and final processed data have been deposited in a public database such as GEO.

☒ Confirm that you have deposited or provided access to graph files (e.g. BED files) for the called peaks.

Data access links
May remain private before publication.

Short read data of ChIP-seq is publicly available at NCBI Sequence Read Archive under accession number PRJNA870030:
https://www.ncbi.nlm.nih.gov/sra/?term=PRJNA870030

Processed ChIP-seq files describing sequencing depth across the genome can be found at:

Liu, Chang (2023). The plant nuclear lamina disassembles to regulate plant genome folding in stress conditions. figshare. Dataset. https://doi.org/10.6084/m9.figshare.21370560.v1

Files in database submission

In the above-mentioned figshare link, the BigWig files of each datasets are available for downloading:
CRWN1_IP_heat_rep1.bw
CRWN1_IP_mock_rep1.bw

CRWN1_input_heat_rep1.bw
CRWN1_input_mock_rep1.bw
CRWN4_IP_heat_rep1.bw
CRWN4_IP_mock_rep1.bw
CRWN4_input_heat_rep1.bw
CRWN4_input_mock_rep1.bw
CRWN1_IP_heat_rep2.bw
CRWN1_IP_mock_rep2.bw
CRWN1_input_heat_rep2.bw
CRWN1_input_mock_rep2.bw
CRWN4_IP_heat_rep2.bw
CRWN4_IP_mock_rep2.bw
CRWN4_input_heat_rep2.bw
CRWN4_input_mock_rep2.bw

The following file describe enriched ChIP-seq peaks:
ChIP_seq_peaks_all.xlsx

Genome browser session (e.g. UCSC)

not available

Methodology

Replicates

2 biological replicates

Sequencing depth

All samples have single-end reads (length 150bp).
Sample name; mapped reads; PCR-duplicate free:
CRWN1_input_mock_rep1; 31068333; 26844135
CRWN1_IP_mock_rep1; 13587630; 11119271
CRWN1_input_heat_rep1; 30090107; 25772785
CRWN1_IP_heat_rep1; 14788386; 11796139
CRWN1_input_mock_rep2; 24008719; 21362757
CRWN1_IP_mock_rep2; 14342205; 11769382
CRWN1_input_heat_rep2; 27634007; 24263484
CRWN1_IP_heat_rep2; 15219102; 12206005
CRWN4_input_mock_rep1; 21551469; 18623181
CRWN4_IP_mock_rep1; 11708589; 9329338
CRWN4_input_heat_rep1; 29452996; 24750951
CRWN4_IP_heat_rep1; 14221913; 11281890
CRWN4_input_mock_rep2; 19802775; 17398652
CRWN4_IP_mock_rep2; 10637104; 8859908
CRWN4_input_heat_rep2; 22541676; 19493653
CRWN4_IP_heat_rep2; 10324096; 8307021

Antibodies

Pierce Anti-HA Magnetic Beads(Catalog number: 88836)

Peak calling parameters

Same parameters were applied for peak calling on different samples. The following command line is for CRWN1 under heat stress:
macs2 callpeak -t IP_heat_1_1.fq_mapped_PE_sorted.bam IP_heat_2_1.fq_mapped_PE_sorted.bam -c input_heat_1_1.fq_mapped_PE_sorted.bam input_heat_2_1.fq_mapped_PE_sorted.bam --broad -f BAMPE -B -g 1.2e8 --bw 450 -n heat --keep-dup=1 --outdir=macs2_c1_cmb_heat

Data quality

Reproducibility between replicates was confirmed by PCA (please see supplemental Figure S4 in the manuscript)

Software

Analyses downstream of peak calling were done with customized scripts in R.
